# Functional paper-based materials for diagnostics

**DOI:** 10.1007/s40828-021-00139-w

**Published:** 2021-03-31

**Authors:** Laura M. Hillscher, Valentina J. Liebich, Olga Avrutina, Markus Biesalski, Harald Kolmar

**Affiliations:** 1 Department of Macromolecular and Paper Chemistry, Technische Universität Darmstadt, 64287 Darmstadt, Germany; 2 Merck Lab @ TU Darmstadt, Technische Universität Darmstadt, 64287 Darmstadt, Germany; 3 Department of Applied Biochemistry, Technische Universität Darmstadt, 64287 Darmstadt, Germany

**Keywords:** Functional papers, point-of-care diagnostics, cellulose functionalization, μPAD, biosensor, lateral flow assay

## Abstract

Functional papers are the subject of extensive research efforts and have already become an irreplaceable part of our modern society. Among other issues, they enable fast and inexpensive detection of a plethora of analytes and simplify laboratory work, for example in medical tests. This article focuses on the molecular and structural fundamentals of paper and the possibilities of functionalization, commercially available assays and their production, as well as on current and future challenges in research in this field.

**Graphic abstract:**

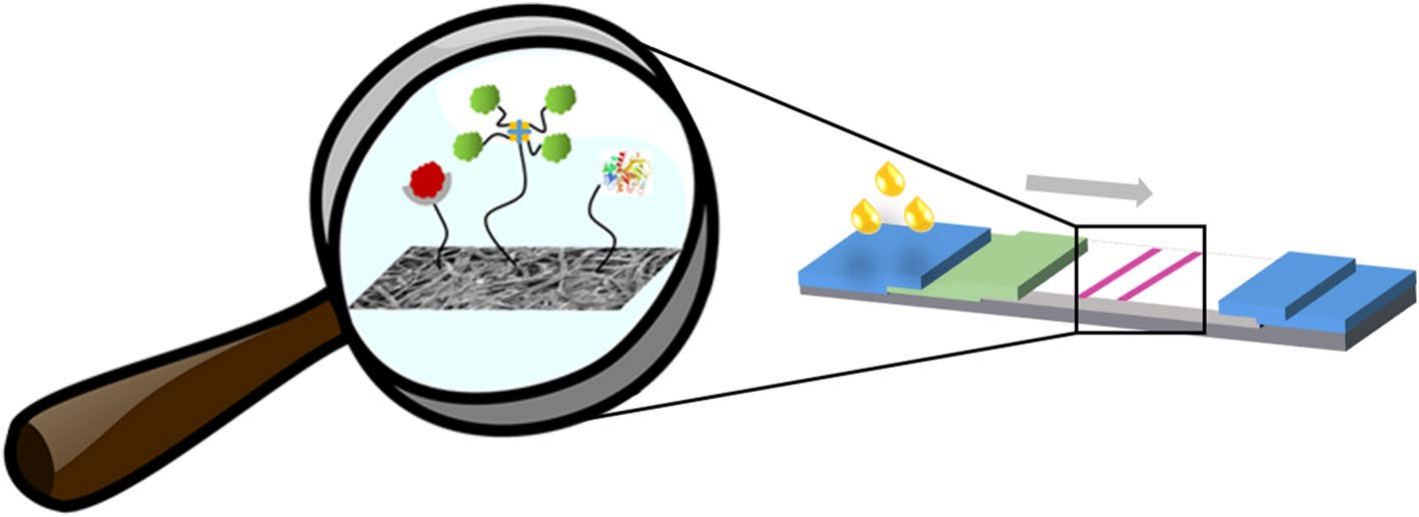

## Introduction

Paper-based sensors are highly attractive not only for researchers. Being very low in cost, easy-to-use, and able to give, in the simplest case, a yes/no answer on questions arising in everyday life, paper-based analytical devices have found their way into all of our households [1].

A prototype of a paper-based urine test to assess the presence of glucose and protein was described by George Oliver, a physician from England, in 1883. However, the first paper dipstick aimed at specific testing for glucose, named Clinistix, was marketed in 1956 by Ames (later Bayer). Intensive research and development efforts have been made since then. Nowadays, it is even possible to test for up to ten biomarkers from just a few drops of urine within a minute using a single multitest dipstick [2].

The same decade when the first commercial dipstick test appeared on the market also provided the onset for today’s commonly known lateral flow assay format, another important type of paper-based analytical device. Only 30 years later, in the 1980s, was this test strip available, e.g., as a convenient home pregnancy test, having marked a real revolution by that time. Since then, over 500 patents have been filed on the development of this technology [3].

Together with recently established diverse types of microfluidic paper-based analytical devices (µPADs), functional papers provide a broad methodological basis addressing a vast variety of analytical questions [4].

General interest in paper-based sensors is unbroken. The global biosensor market will probably grow by more than 70% by 2022, compared to 2015 [5]. Improvement of existing and design and implementation of new, low-cost, and user-friendly point-of-care devices are being cited as the main drivers of this development. Obviously, paper-based technologies have the potential to meet most of the ASSURED guidelines filed by the World Health Organization (WHO) for diagnostics in resource-constrained settings. According to the WHO, such tests must be Affordable, Sensitive, Specific, User-friendly, Rapid and robust, Equipment-free, and Deliverable [6].

In this respect, cellulose and its derivatives feature several promising properties and offer some evident benefits. Present in practically all plants, this natural product is abundantly available and therefore inexpensive [7]. It is environmentally sustainable, highly bioavailable, biodegradable, and biocompatible. Cellulose is insoluble in the majority of common organic solvents, in particular in water, and is able to transport water-soluble substances just by capillary forces, without further employment of an external power source. Moreover, substances may be stored inside the paper pores [8]. Finally, paper is chemically and thermally stable, easy to handle and store, and safely disposable or even recyclable, if desired [8, 9]. Taken together, these properties caused a still growing interest in paper-based analytical devices [9].

Regardless of the number of interesting demonstrations reported to date, a number of challenges still exist. In particular, if paper samples are used as a materials source for medical diagnostic tools. There are already numerous valuable review articles available on the newest developments on such applications. However, stepping one step further towards any successful transfer of such demonstrations into market-ready devices requires a more in-depth look at the paper material and its structure–property relationship itself. The latter progresses from looking at essential building elements of paper, the paper lignocellulosic fiber, to papermaking technology, including ways of controlling the porous inner structure, the ability to functionalize paper fibers in a tailored fashion, and finally to integrate such highly complex and functional biogenic materials into paper-based sensor devices. In order to address these points, this article will start out with looking at the historical, yet innovative papermaking process, followed by looking in detail at the chemical and geometrical structure of fibers being used in papermaking. Then we will focus attention on the chemical properties of cellulose, as the major building block of paper fibers, as well as its different opportunities and challenges for modifications as a basis for the further design of paper-based sensors. Finally, will focus on commercially available paper-based sensors, especially their setups and techniques. Here, the latest developments and their potential will be presented and discussed, as well as remaining challenges, requirements, and future directions.

## Introduction to paper

### History, manufacture, and current use

For almost 2000 years, paper has been defined as a porous, flat, non-woven, layered material made of mostly natural fibers. Commonly, such a paper sheet is made through dewatering of a fiber suspension on a sieve and subsequent drying under compression and thermal heating of the resulting fiber fleece. This fiber fleece is composed of many statistically and randomly aligned individual fibers, constituting a highly porous organic material [10–14].

Historically, the invention of paper goes back to the year 105 ad. The Minister of the Chinese Imperial Court Cai Lun used old textiles and mulberry bast to make paper as a writing material, which is nowadays known as the birth of paper. With the takeover of Turkmenistan by the Arabs, paper finally reached Europe via Arabia around 760. The oldest existing European paper document dates back to 1102, and the first paper mill that started producing paper in Germany was in Nuremberg in 1390. As a result of the invention of letterpress printing by Johannes Gutenberg in 1435, the demand for paper experienced a strong increase [11–13, 15].

**Fig. 1:**
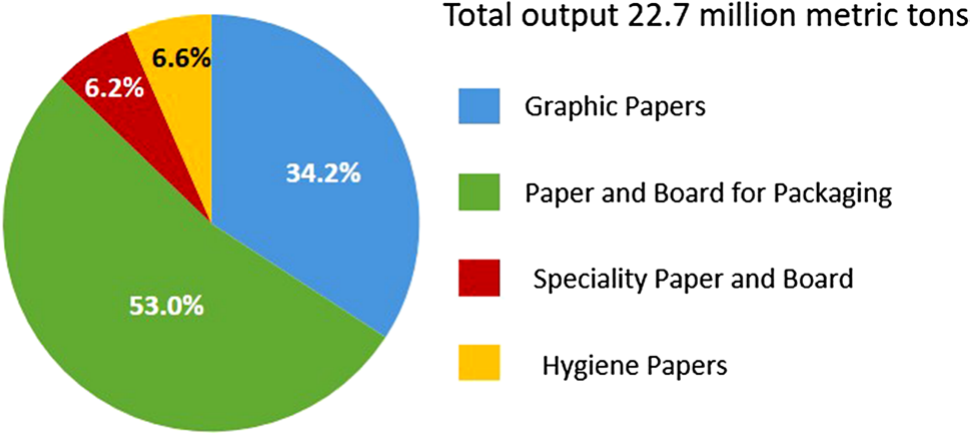
Shares of the individual paper grades in the total annual paper production in Germany in 2018. [17]

Worldwide, approximately 420 million metric tons of paper are produced annually, the largest paper-producing countries being China, the USA, Japan, and Germany (as of 2018) [16]. The areas of paper application are manifold and range from graphic papers, packaging papers, hygiene papers to special papers for, e.g., diagnostic, decor, or filter applications. The shares of the individual paper grades in the total annual paper production in Germany are shown in Fig. 1. Packaging papers account for the largest share, followed by graphic papers, hygiene papers, and the smallest share for special papers. Nevertheless, this segment should not be underestimated, as its 6.2% market share still corresponds to approximately 1.4 million metric tons of annual production and is sold at significantly higher prices than the papers of the other three grades [17]. The functional papers presented in this review also all belong to the category of special papers.

At a first glance, paper seems to be a simple and scientifically less challenging, historic material. Up to now, research on paper has therefore mainly focused on the processing and the properties of the material with respect to its standard applications, progressing from packaging material to print media and hygiene products. The necessity of meeting our needs in the future, to a large extent from renewable resources, calls for further use of paper as a biogenic, recyclable material, beyond its classic fields of application. Paper can be functionalized in a variety of ways. This feature allows the production of functional paper-based materials for sensor technology, microfluidics, and a variety of other high-tech applications. With respect to synthesis and analysis, it is a tremendous challenge to capture the complexity of the material over all length and time scales. However, the latter is perhaps the most important key to tailor the geometric structure and chemical composition of functional paper.

Paper properties are highly versatile and can differ greatly in terms of fiber type and origin, production process, fillers and additives used for papermaking, and finally on any kind of refinement and functionalization. Table 1 summarizes the properties of three different commercially available Whatman® filter papers. The most outstanding property of paper is that it consists primarily of cellulose fibers and thus of the world’s most abundant raw material. It is estimated that more than 100 billion tons of cellulose are produced annually by nature via biosynthesis, mainly by plants of all species, but there are also some bacteria which are capable of producing cellulose [18–20].

**Table 1:** Properties of commercially available Whatman® filter papers [21, 22]

Paper type	Thickness (µm)	Basis weight (g/m^2^)	Porosity
Whatman #1	180	87	0.707
Whatman #4	210	92	0.736
Whatman #5	200	100	0.702

The basic building blocks of paper are single lignocellulosic fibers of natural origin or of recycled fibers, so-called secondary fibers. Natural fibers are obtained directly from plant raw materials by pulping. Pulping describes the process of disintegration of the raw material wood into fibers and can either be carried out mechanically or chemically. Mechanical pulping separates fibers from woodchips by applying strong mechanical shear forces. Among other things, a rotating millstone is used for this purpose, which presses and shears the wood surface, thereby separating out individual fibers. Paper fibers obtained from this pulping process still contain a larger amount of lignin as well as hemicelluloses. The latter can affect the behavior of such fibers in contact with water, as well as the chemical composition of the surface of such fibers which can be very different from that of cellulose itself [23, 24]. Chemical pulping, on the other hand, dissolves lignin and other components of the interfiber matrix material, thus separating the individual fibers, which then typically contain only low amounts of the aromatic and nonpolar compound lignin from the rest of the wood-based materials. The pulping process, the raw material, and its origin therefore all have a strong influence on the fiber properties. For example, fibers still containing nonpolar lignin or other hemicellulosic material may absorb water and swell to a different degree than those fibers that are composed of pure cellulosic polymers. The latter may be of great importance in the design of paper-based sensors that are in contact or transporting aqueous analyte-containing fluids.

Worldwide, 90% of the raw material source is wood. Depending on the wood source, the obtained fibers moreover differ in length and stiffness, which will also impact the final mechanical properties of the resulting paper, and as such its use in different applications. In addition, pulp from jute, flax, hemp, sisal, grasses, and cotton is also used in paper production [12, 13, 25]. In particular, paper produced from cotton and cotton linters fibers is predominantly used for the design of functional papers in biomedical or other highly specialized applications. The latter is due to their high purity, related to a high cellulose content in the fibers, which far exceeds 95% [24–27].

### Paper fiber morphology

**Fig. 2:**
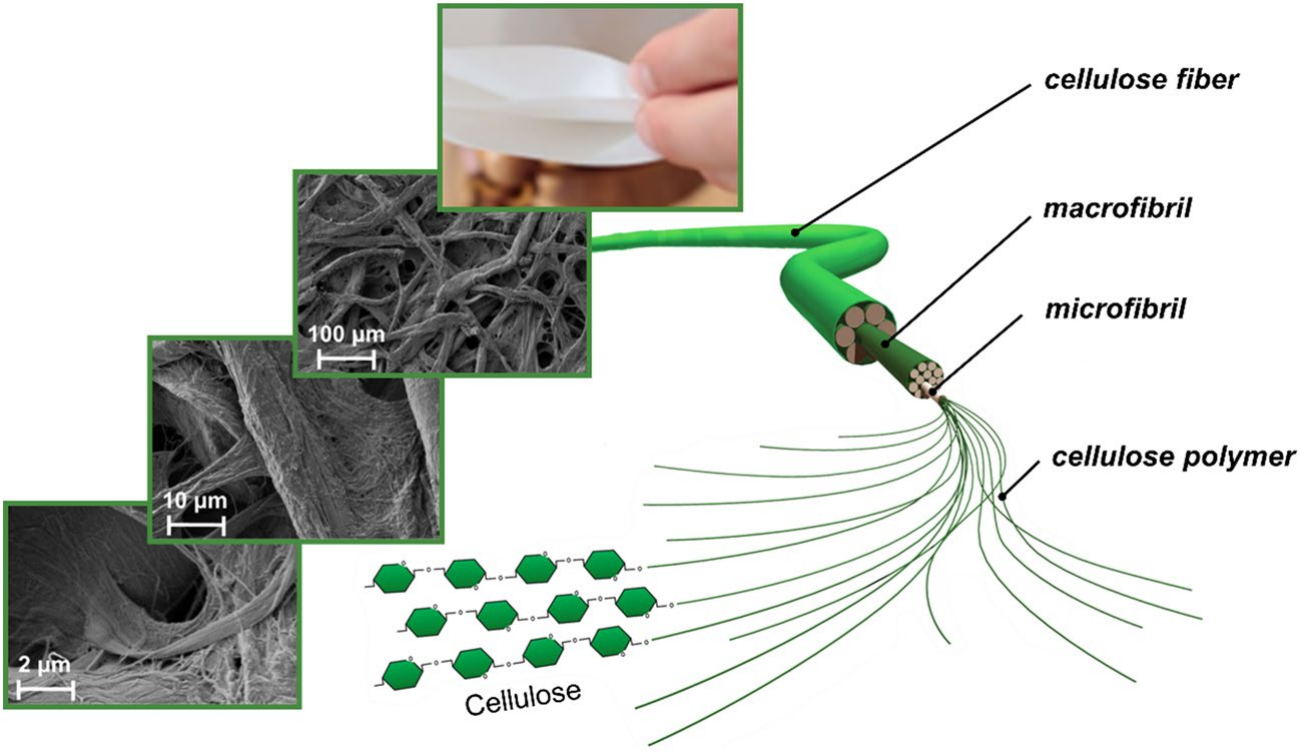
Supramolecular structure of cellulose fibers. Reproduced with permission from [29], electron microscopy pictures with permission from [30]

In a simplified perspective, a fiber can be thought of as a straw consisting of the fiber wall enclosing a cavity called the lumen. Chemically, fibers are composed of three main components, cellulose, hemicellulose, and lignin. Higher up in the morphological hierarchy, these components form macromolecules, each performing different tasks in the fiber composite. Cellulose serves as framework material, hemicellulose acts as matrix and embedding material, and lignin as an encrusting material, giving mechanical strength to the cell wall. A simplified picture of the morphological constitution and substructure of a wood fiber can be seen in Fig. 2. The cellulosic polymer strands are ordered into elementary fibrils with a size of 3–35 nm. These are ordered into microfibrils in the next morphological hierarchy, which are in the size range of 10–35 nm. In a hierarchical fashion, microfibrils form larger macrofibrils that are embedded between hemicellulose and lignin. All these morphological structures are stabilized by hydrogen bonds between cellulose units for elementary, micro- and macrofibrils, as well as for the interface bonding between cellulose, hemicellulose, and lignin [25, 28].

### Chemical and supramolecular structure of cellulose

**Fig. 3:**
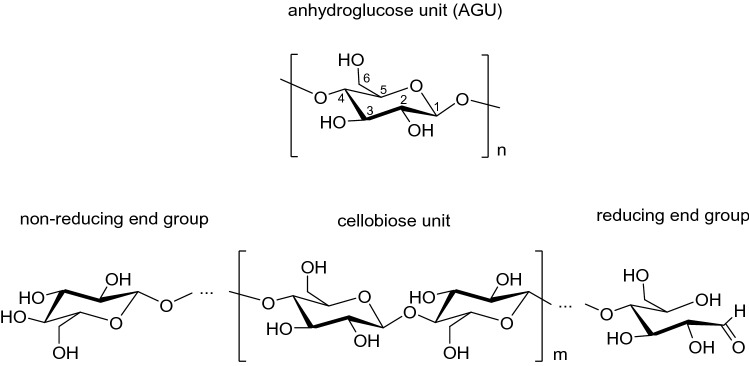
Chemical structure of anhydroglucose (AGU), cellobiose, and the non-reducing and reducing end groups of a cellulose polymer chain

Cellulose is a linear polymer composed of β-(1,4)-glycosidically linked d-anhydroglucopyranose units (AGUs). The dimer of two AGUs forms cellobiose, which is often referred to as the monomer building block of cellulose. A cellulose polymer chain has two different end groups. A non-reducing end group comprises an intact glucose ring with a free hydroxyl group at the C4 atom, and a reducing end group, which can expose an aldehyde group at C1 in equilibrium with the corresponding closed-ring hemiacetal OH group. As a result of the β-glycosidic linkages, the cellulose polymer exhibits a linear, fully extended secondary structure. In Fig. 3, the chemical structures of AGU, cellobiose, and the reducing and non-reducing end groups are depicted. Typically, the degree of polymerization (DP), which is the number of monomeric units, is in the range of 300–1700 AGUs for wood pulp and around 800–10,000 AGUs for cotton and other plant fibers. Historically, the polymeric structure of cellulose, as known today, was first elucidated by Hermann Staudinger in 1920 and thus laid the foundation for modern polymer sciences. He proved that cellulose is not a noncovalent arrangement of glucose units, but rather consists of covalently linked repeating blocks to form a linear macromolecule [18, 31].

**Fig. 4:**
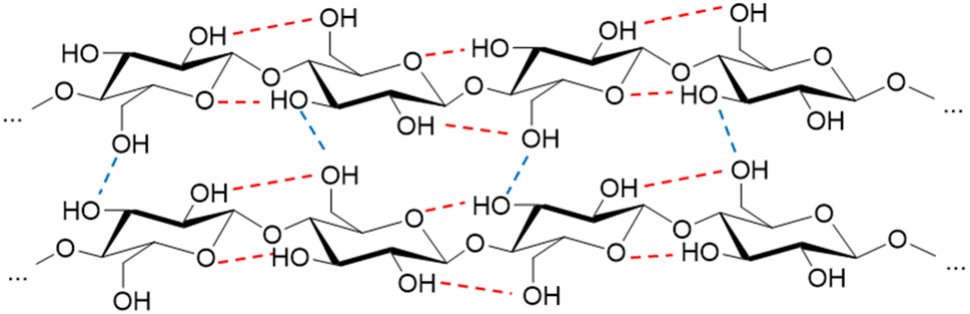
Section of the supramolecular structure of native cellulose. Intermolecular hydrogen bonds are indicated in blue and intramolecular hydrogen bonds in red. Reproduced with permission from [29, 32]

Regarding the chemical structure, it is obvious that cellulose consists purely of carbon, hydrogen, and oxygen atoms. Therefore, it belongs to the subgroup of carbohydrates. As a result of its numerous hydroxyl groups enabling the formation of hydrogen bonds and the linear conformation of the cellulose polymer chains, cellulose possesses a dense and strongly cohesive arrangement. Intermolecular hydrogen bonds join individual cellulose polymer chains together, whereas the linear conformation of each individual cellulose chain is stabilized by intramolecular hydrogen bonds. A section of the resulting supramolecular structure with the according hydrogen bond network is depicted in Fig. 4.

**Fig. 5:**
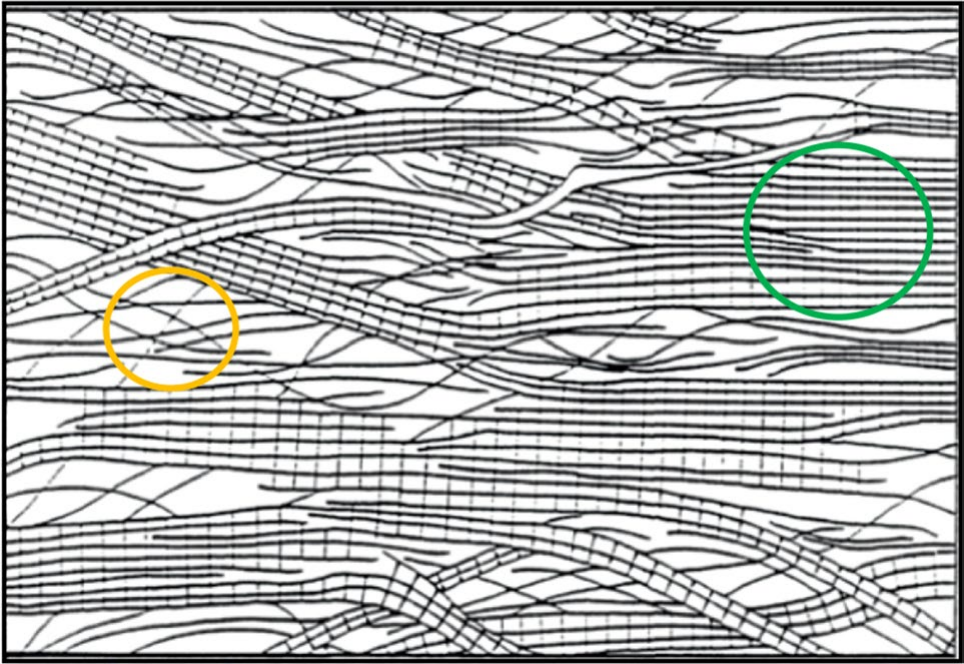
Fringed fibril model of the supramolecular structure of cellulose, as described by Haerle in 1958. An exemplary crystalline region is indicated in green and an exemplary amorphous region in yellow. Reproduced with permission from [28]. Copyright (1998) Wiley-VCH GmbH

Additionally, the supramolecular structure of cellulose is composed of highly ordered, crystalline and disordered, amorphous regions. The crystalline ones consist of the highly linear aligned cellulose chains, with a network of strong hydrogen bonds, as shown in Fig. 4. Besides these crystalline regions, there are also less-ordered, amorphous ones where the cellulose polymer chains are not linearly aligned. Figure 5 shows the *fringed fibril model* describing the supramolecular structure of cellulose that was developed by Haerle in 1958. It demonstrates the different constitutions of ordered crystalline and disordered amorphous regions [28, 33]. For the mechanical properties of a fiber, the crystalline regions provide strength and stiffness, while the amorphous domains ensure elasticity and flexibility [34].

**Fig. 6:**
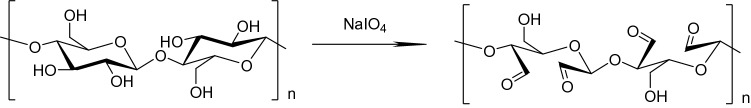
Sodium periodate oxidation of cellulose leading to 2,3-dialdehyde cellulose

Owing to the strong cohesive and semicrystalline structure, cellulose is chemically very stable and insoluble in water or other common organic solvents [28]. However, paper fibers can swell in contact with aqueous solutions, and can take up in equilibrium 8–14 wt% of water [24]. Water can solely penetrate into the amorphous cellulose areas, where it interferes with the intra- and intermolecular hydrogen bonds, thus increasing the total volume of the fiber. Concentrated sodium hydroxide solutions can even penetrate into the crystalline cellulose regions because of the strong interactions between the dissociated hydroxyl ions and the hydroxyl groups of cellulose. This process leads to variations in the supramolecular structure, including conformation changes, and is often non-reversible. In contrast, cellulose hardly swells in most organic solvents, excluding DMSO or ethanolamine, which even cause cellulose to swell more than in water. In general, cellulose swelling depends on the strength of hydrogen bonds, as well as on the polarity of the surrounding liquid. For the same reason, cellulose cannot melt, but decomposes at temperatures of 200 °C and above [28]. In contrast, exposing cellulosic paper materials to periodate causes them to partially degrade or geometric deformations, such as buckling and other non-linear conformational changes, ultimately resulting in cellulose fiber shrinkage [35, 36]. In particular, periodate oxidation causes cleavage of the accessible cellulose rings, as well as the formation of intramolecular hemiacetal bonds between aldehyde groups and the primary alcohol in 2,3-dialdehyde cellulose (Fig. 6).

### Cellulose functionalization

In general, cellulose offers numerous possibilities for functionalization owing to the large amount of addressable hydroxyl groups. Indeed, each AGU has two secondary hydroxyl groups at C2 and C3 and one primary hydroxyl group at C6. These three hydroxyl groups per AGU exhibit different reactivities, depending on the type of reaction, the reaction solvent and environment, as well as on other factors.

We have already pointed out that cellulose is insoluble in most common solvents; nevertheless, some special solvent systems are capable of dissolving the macromolecule. Thus, two different types of cellulose functionalization reactions are generally possible, homogeneous, single-phase reactions in the dissolved state or heterogeneous, two-phase reactions on intact cellulose fibers. Homogeneous reactions lead to a highly uniform cellulose functionalization and distribution of the substituents along the cellulose polymer chains. In heterogeneous reactions, on the other hand, mostly just the amorphous and swellable cellulose parts are converted. In general, homogeneous functionalization under disruption of the supramolecular cellulose structure, combined with a change in the chemical structure, usually leads to products that can be hardly compared to the cellulosic starting material. In the following, the most common and important cellulose functionalization reactions are explained in detail.

Chemical cellulose functionalization is primarily based on the so-called polymer analogue reactions on hydroxyl groups. A polymer analogue reaction is principally defined as a chemical reaction on a polymer with preservation of the polymer backbone. In case of cellulose, three hydroxyl groups, namely one primary at the C6 position and two secondary ones at C2 and C3, are available. As mentioned above, these hydroxyls are all involved in the strong hydrogen bond network of cellulose. In general, the OH groups at C2 and C6 have multiple reaction possibilities compared to the hydroxyl group at C3, as they are statistically less involved in the hydrogen bond network, which in turn leads to higher chemical reactivity. The primary hydroxyl group on C6 is also more reactive than the secondary hydroxyl group on C2 for the same reason. Additionally, the steric accessibility of the hydroxyl groups also has an influence on functionalization reactions. In two-phase reactions, solely the amorphous regions of cellulose are accessible, whereas in homogenous ones nearly all hydroxyl groups are reactive. To enhance the accessibility of hydroxyl groups in a two-phase reaction, different chemical and mechanical treatments can be conducted, e.g., amplification of fiber fibrillation by fiber beating, or the increased swelling of fibers in sodium hydroxide (NaOH) [32, 37]. However, it is obvious that the maximum degree of substitution (DS), which describes the number of chemically converted hydroxyl groups per AGU, cannot exceed three.

Functionalization reactions on cellulose have been the focus of some scientific research for many years [18, 38–40]. Therefore, a wide range of reactions have been reported to date. In the scope of this review, functional papers for diagnostic and sensory applications will be discussed in more detail; hence, strategies for cellulose functionalization, which are important in this particular field of application, will be addressed in the following.

**Fig. 7:**
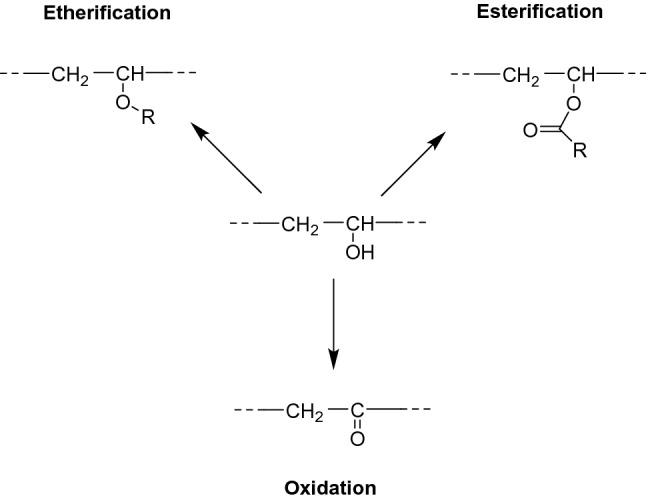
Scheme of typical polymer-analogue functionalization reactions on hydroxyl side groups

The most common polymer-analogue reactions on hydroxyl groups are shown in Fig. 7. Technically, such esterification and etherification are the most important functionalization reactions of cellulose. These derivatives are among others utilized in the food, pharmaceutical, and cosmetics industry [18, 32, 37, 40, 41]. With regard to functional papers, cellulose nitrate (also called nitrocellulose) is the most important cellulose ester derivative. Generally, cellulose esters are produced by esterification of the cellulose hydroxyl groups with organic acids, mainly in the presence of strong inorganic acids. Nitrocellulose is formed through esterification with nitric acid (HNO_3_) in the presence of strong inorganic acids, e.g., sulfuric acid (H_2_SO_4_). Owing to its exceptionally good protein binding properties, nitrocellulose is used as a substrate for most lateral flow immunoassays (LFIA), with the home-use pregnancy test as an example of a commonly known and commercially available system [32, 37].

Cellulose ethers are obtained according to the commonly known Williams ether synthesis, by converting the hydroxyl groups of cellulose with alkyl halides in the presence of strong bases. A prominent example of a widely used cellulose ether derivative is carboxymethyl cellulose (CMC). CMC is produced by etherification of the cellulose hydroxyls with monochloroacetic acid in the presence of NaOH, and CMCs are utilized, for instance, in the textile industry as a coating for yarns or as binding and thickening agents in the food industry [28, 37]. Concerning functional paper applications, CMCs offer the advantage of exposing free carboxyl groups that can be easily used for subsequent immobilization reactions [42].

**Fig. 8:**
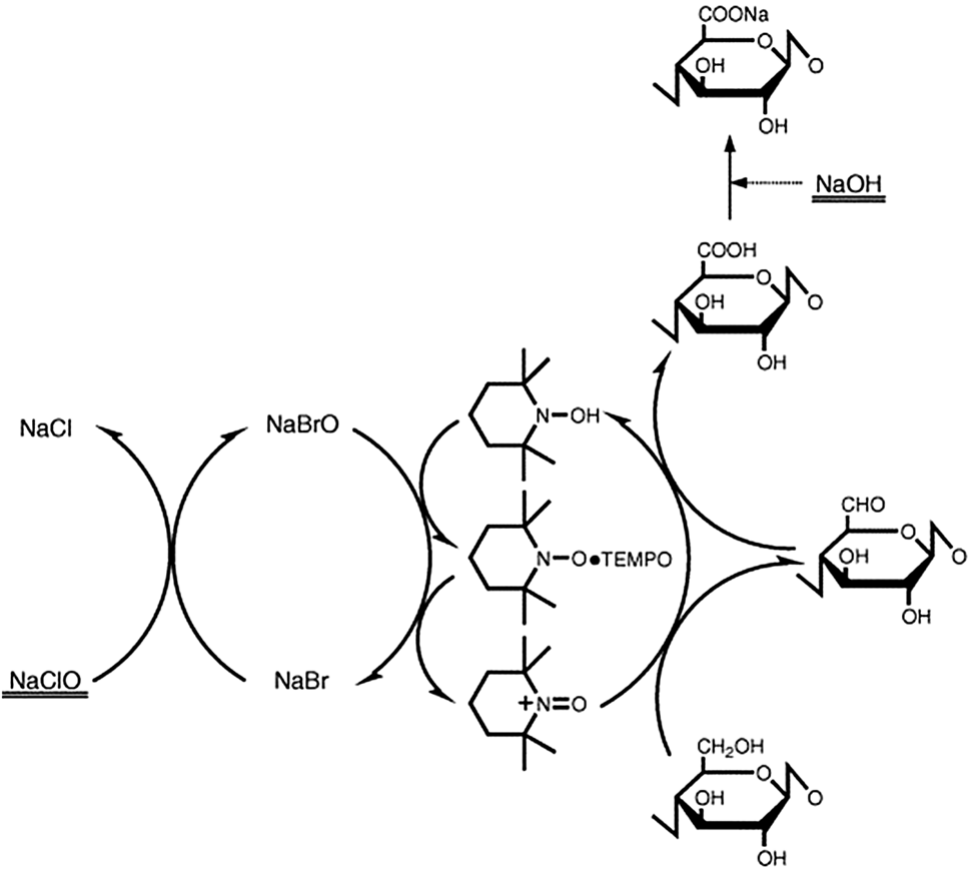
Reaction scheme of TEMPO-mediated cellulose oxidation. Reproduced with permission from [45]. Copyright (2006) Elsevier

For the same reason, cellulose oxidation is another frequently conducted modification to increase the carbonyl or carboxyl content, with periodate and TEMPO-mediated reactions being the most common examples. In the former approach, the secondary hydroxyl groups on C2 and C3 of cellulose are oxidized using sodium periodate (NaIO_4_), leading to an increased carbonyl content. This reaction is accomplished by opening of the cellulose pyranose ring, evoking damage to the cellulose polymer chain, followed by a change of its supramolecular structure [37, 43, 44]. During the TEMPO-mediated cellulose oxidation, the primary C6 hydroxyl groups are transformed to carbonyls and, further, to carboxylate groups as shown in Fig. 8. Hence, the oxidation of the hydroxyl group on the C6, which is not part of the pyranose ring, maintains the polymer backbone intact. The TEMPO-mediated cellulose oxidation is conducted in aqueous alkaline medium and uses 2,2,6,6-tetramethylpiperidinyloxyl (TEMPO) as catalyst, sodium bromide (NaBr) as co-catalyst, and sodium hypochlorite (NaOCl) as oxidant [37, 45–48].

## Biofunctionalization of paper

**Fig. 9:**
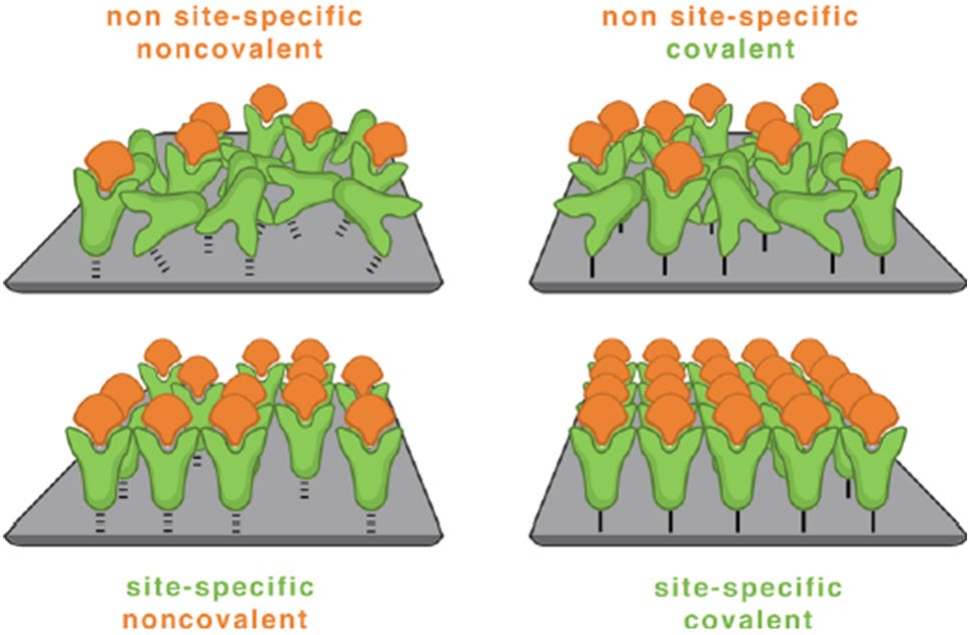
Schematic of the four different immobilization methods for biomolecules on surfaces. From top left to bottom right: non-site-specific, noncovalent immobilization by physical adsorption; non-site-specific, covalent immobilization by covalent binding; site-specific, noncovalent immobilization by bioaffinity coupling; and site-specific, covalent binding by bioorthogonal coupling. Note, the dashed lines between the green antibodies and the surface (upper and lower left schemes) represent weak physical interactions, whereas the solid lines (upper and lower left schemes) represent covalent strong interactions between the surface and the antibody, respectively. Reproduced with permission from [49]. Copyright (2013) American Chemical Society

The working principle of paper-based biosensors is predicated on the binding of target molecules from the sample to be analyzed to their corresponding binding partners, which are immobilized on the paper substrate, leading to a readout signal. For optimal binding efficiency and specificity, the biomolecule’s orientation on the paper substrate and its full coverage are crucial. Generally, four different principles for biomolecule immobilization, namely physical adsorption, covalent coupling, bioaffinity, and bioorthogonal coupling, are feasible. These four methods can be divided into two different subgroups, the site-specific (oriented) and non-site-specific (random) methods. As depicted in Fig. 9, physical adsorption and covalent coupling both belong to the non-site-specific group, whereas bioaffinity and bioorthogonal coupling both belong to the group of site-specific methods. In fact, physical and chemical coupling methods lead to a non-site-specific immobilization, whereas biochemical coupling methods lead to a site-specific immobilization of the biomolecule [37, 49–52].

Among the four immobilization methods, biomolecule immobilization on paper by physical adsorption is the most straightforward and frequently used technique. Thereby, the immobilization is conducted by simply dipping paper into a biomolecule-containing solution, or coating and printing this solution onto paper with a subsequent washing step. Generally, no further pre-/post-treatments procedures are necessary. Hence, coupling is assured by hydrogen bonds, electrostatic/ionic interactions, van der Waals forces, or hydrophobic interactions between the paper substrate and the biomolecule. The main advantage of this immobilization technique is the simple coupling procedure, whereas the disadvantage is the relatively weak and non-site-specific binding [49–52].

**Fig. 10:**
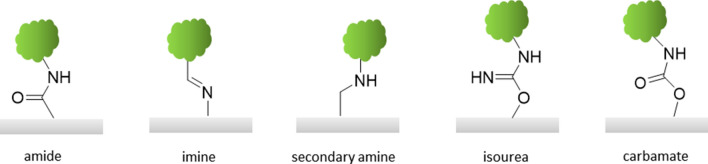
Chemical nature of covalent bonds between model substrates (gray) and biomolecules (green). Reproduced with the permission from the Royal Society of Chemistry from [37]. Copyright (2013) American Chemical Society

The covalent immobilization represents a more permanent and robust method, compared to the noncovalent ones. It relies on a chemical reaction between two functionalities—one on the biomolecule and another on the paper substrate—resulting in a covalent linkage. The nature of the chemical reactions is limited to those that can be conducted in a physiological environment (aqueous buffers at around neutral pH) to avoid denaturation of the biomolecule. Commonly, functional groups present in biomolecules, among them amines, carboxyls, hydroxyls, and thiols, are used as reaction partners. On the other hand, functional groups available for immobilization on paper are limited to poorly reactive hydroxyls, for which reason they must be chemically activated in advance. As mentioned above, a commonly used activation method is the TEMPO-mediated oxidation that converts the primary cellulosic hydroxyl on C6 (Fig. 8). The emerging aldehyde and carboxyl groups are reactive enough to be used for subsequent coupling reactions. Prominent examples of chemical reactions used for covalent immobilization of biomolecules on paper substrates include amination catalyzed by *N*-hydroxysuccinimide esters (NHS) in combination with 1-ethyl-3-(3-dimethylaminopropyl)carbodiimide hydrochloride (EDC) or imination through condensation of primary amines with aldehyde moieties. The most common functional groups in biomolecules and their potential reaction partners on the paper substrate are summarized in Table 2 and Fig. 10. All covalent immobilization methods combine the advantage of being more robust than physical methods, but are rather complex compared to physical immobilization and as well non-site-specific [37, 49].

**Table 2:** Functional groups available in proteins and corresponding surface functionalities required for coupling [37]

Side-chain group	Amino acid	Surface functionalities
–NH_2_	Lysine	Carboxylic acid, active ester, epoxide, aldehyde
–SH	Cysteine,	Maleimide, pyridyl disulfide, vinyl sulfone
–COOH	Aspartic acid, glutamic acid	Amine
–OH	Serine, threonine	Epoxide

**Fig. 11:**
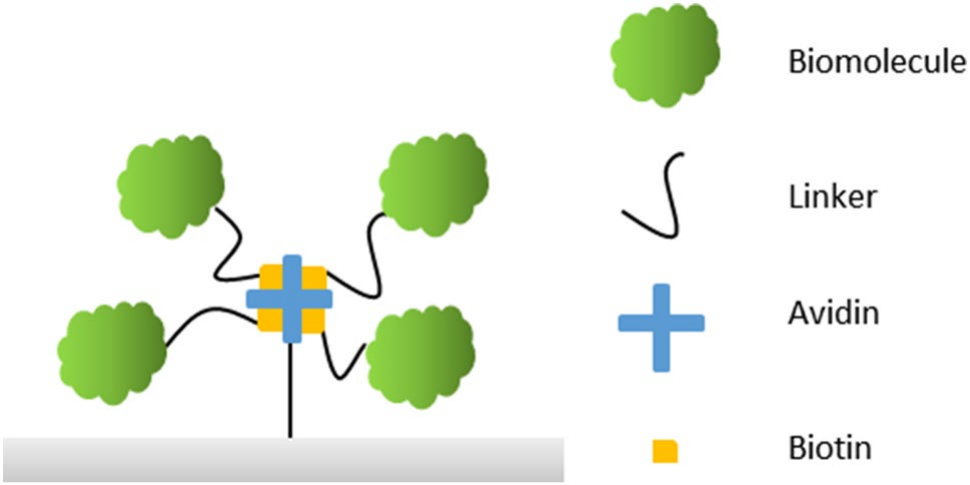
Bioaffinity-based immobilization of a biomolecule (green) an a model substrate (gray) using avidin (blue) and biotin (yellow)

In addition, bioaffinity-based immobilization methods ensure a highly specific and oriented coupling between the paper substrate and the biomolecule under fully retained biological activity. They are based on specific complementary affinity interactions, e.g., antibody–antigen or avidin–biotin ones, occurring in almost all biochemical processes. The schematic principle of bioaffinity-based coupling is shown in Fig. 11. However, this method is rather complex and usually requires modification both of the biomolecule and the paper substrate. Despite the rather complex preparations for a bioaffinity-based binding, this method exhibits the advantage of tunability of the biomolecule’s orientation on the paper substrate by wise selection of the functionality and its structural incorporation into the biomolecule. However, bioaffinity-based immobilization belongs to the group of site-specific, noncovalent methods and often leads to a higher biological activity than the physical and covalent methods [37, 49, 53].

**Fig. 12:**
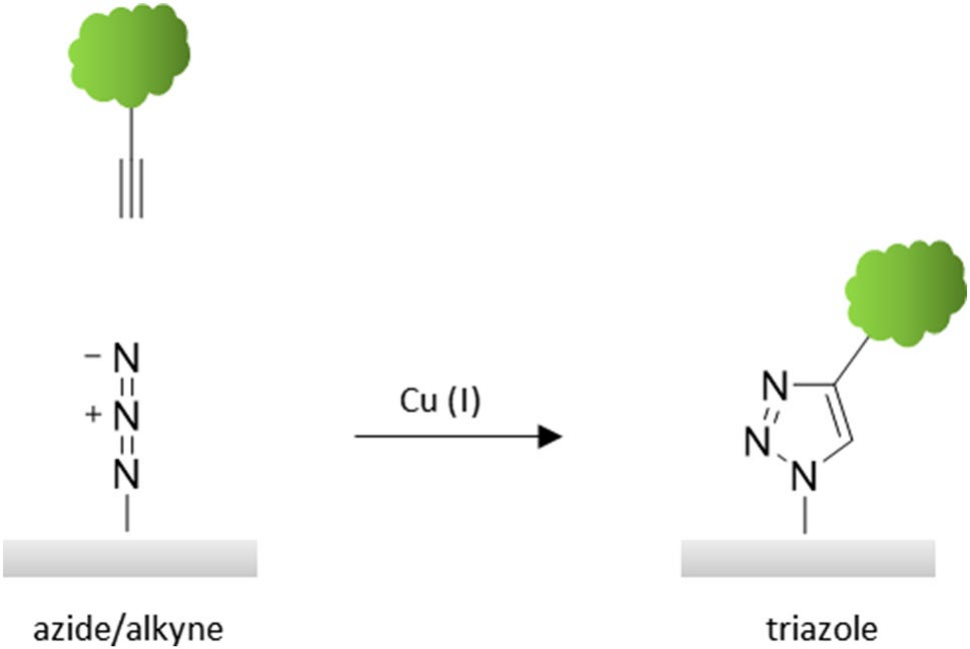
Bioorthogonal biomolecule (green) immobilization via copper-catalyzed azide–alkyne cycloaddition (CuAAC) on a model substrate (gray)

To achieve an oriented and covalent immobilization of the biomolecule on the paper substrate, bioorthogonal immobilization is the method of choice. To meet the two essential prerequisites, specifically reacting chemical functionalities have to be introduced on the paper substrate, as well as on a structurally specific site of the biomolecule. The crucial requirement for bioorthogonal coupling is that the chemical functionalities react specifically with each other without exception, and do not address other functionalities of the biomolecule or the paper substrate. Prominent examples for such biorthogonal chemistries are the so-called click reactions, especially Cu(I)-catalyzed or ring-strain-induced azide–alkyne cycloadditions (Fig. 12). Bioorthogonal immobilization reactions provide the same advantages as bioaffinity-based ones, including site-specific coupling. However, bioorthogonal immobilization leads to a more robust, covalent coupling [37, 49, 54–56]. Recently developed strategies allow for the oriented conjugation of biomolecules on paper using recombinantly produced variants that contain special recognition sequences for bond-forming enzymes such as Sortase A or microbial transglutaminase [57, 58].

## Commercial paper-based assay formats

### General remarks

Analytical devices which are commercially available as of today and which consist in part of cellulosic material rely on two different types of assays, paper test strips and lateral flow assays (LFAs) [59]. Test strips, also called dipsticks, are used for a broad range of applications, from environmental, food and beverage probes to diagnostics. Commonly, they are used to determine or prove pH values, the presence of heavy metals, glucose levels, or peroxidase activity [60]. LFAs, on the other hand, are used in diagnostics, environmental or food safety control. The mixtures to be assessed can be very complex. Besides urine or saliva, even whole blood can be tested [61]. The most prominent LFA is the home-use pregnancy test. Other important applications are, e.g., the detection of pesticides or pathogens in food [60] or a test against anti-SARS-CoV-2 antibodies in human blood samples [62]. The success of paper-based analytical devices on the market is based on the simplicity of the execution and their readout, which can be performed by an untrained user often by naked eye detection.

### Paper test strips

**Fig. 13:**
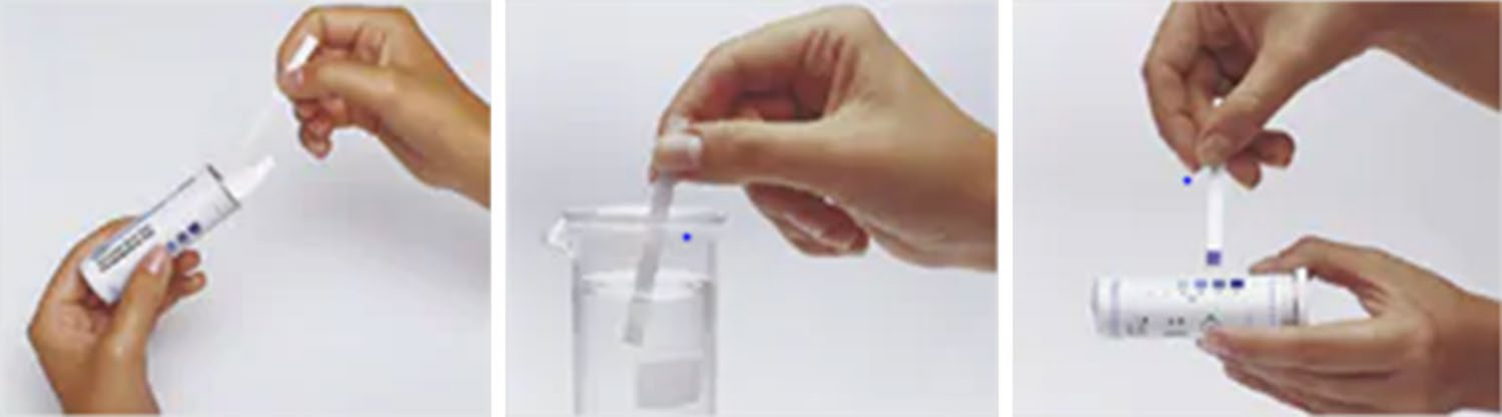
From left to right. A dipstick is taken from the package. The test is performed. The strip is compared to a reference depicted on the package. Reproduced with permission from [65]. Copyright Merck KGaA Darmstadt

Test strips are often described as “dry chemistry”. The chemical reactions in this assay, other than it might imply, cannot take place in the dry state, but the counterparts are readily dissolved upon contact with the sample solution. Paper dipsticks consist of several layers. In their simplest form, one detection zone in the form of filter paper, impregnated with the reagents, is affixed on a non-reacting thermoplastic backing that also serves as handle. When dipped into a sample solution, access liquid is removed and the test result is visible owing to an arising color in the detection zone after incubation within a defined period. For analysis, the emerged color (pattern) must be compared in respect to color shade or intensity to a given color chart. Thereby, a qualitative or semiquantitative result about the presence and, in some cases, also concentration of the analyte is obtained [63]. To ensure uniform distribution of the sample throughout the reaction zone and therefore uniform color development of the strip and to prevent “bleeding”, a mesh, e.g., made of nylon, is used to affix the reagent papers onto the carrier foil [64]. Underlying chemical reactions are versatile, and the resulting dye, as well as the sensitivity of the tests, even for the same analyte, vary according to the test manufacturer. An example procedure of a test strip analysis is given in Fig. 13.

Test strips are user-friendly, inexpensive, and deliver quick results. However, despite all the ease in use of the dipstick assay, they exhibit certain drawbacks. Color can intensify and thereby falsify the test result when incubation times are exceeded. Thus, precise timing is required to obtain semiquantitative results. Additionally, the interpretation of a color is always subjective, so that the result obtained depends on the tester. Besides, the sensitivity and limit of detection are defined by the pore volume of the paper patch, as it limits the applicable sample volume [63].

**Fig. 14:**
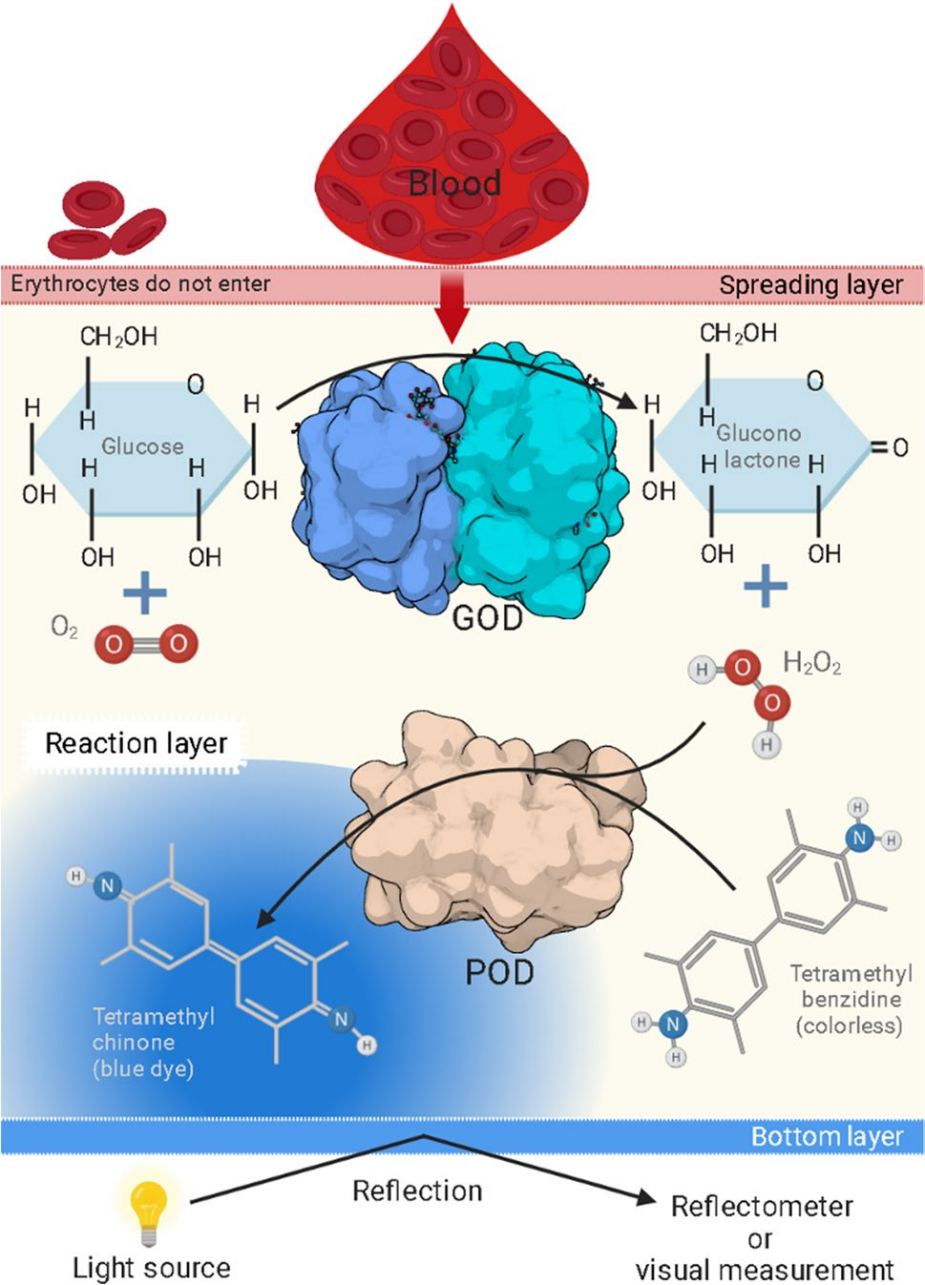
Glucose test strip composition for a bottom readout. Figure created with biorender.com. Protein structures from PDB (GOD: 1GPE; POD: 1HCH). Reproduced from [66]

To meet the challenges concerning consistent readout, more sophisticated test strips were developed that are compatible with a dedicated reader. Those strips consist of a spreading layer on top that is responsible for the sample uptake and its homogenous distribution throughout the reaction zone. This spreading layer is made of cellulose acetate and impregnated with pigments like titanium(II) oxide or barium sulfate to provide light reflection. Owing to its porosity, the spreading layer holds back larger particles, e.g., blood cells, from entering the reaction layer. The reagents themselves are embedded in hydrophilic polymer matrices like gelatin or agarose, which are coated to the backing, if necessary in separate layers, making sure the single reagent layers cannot influence each other. The bottom layer is a transparent polyester plastic backing. From this bottom side, measurement is carried out either visually or with a reflectometer [66]. Figure 14 shows a scheme of such a blood glucose test strip.

Glucose is a very well-known representative to be tested in a dipstick format. It is commonly part of a combined urinalysis dipstick to determine the glucose content in urine for diabetes mellitus screening. But they are frequently used in the food and beverages industry, where the knowledge of glucose levels in raw materials and final products is important [67].

**Fig. 15:**
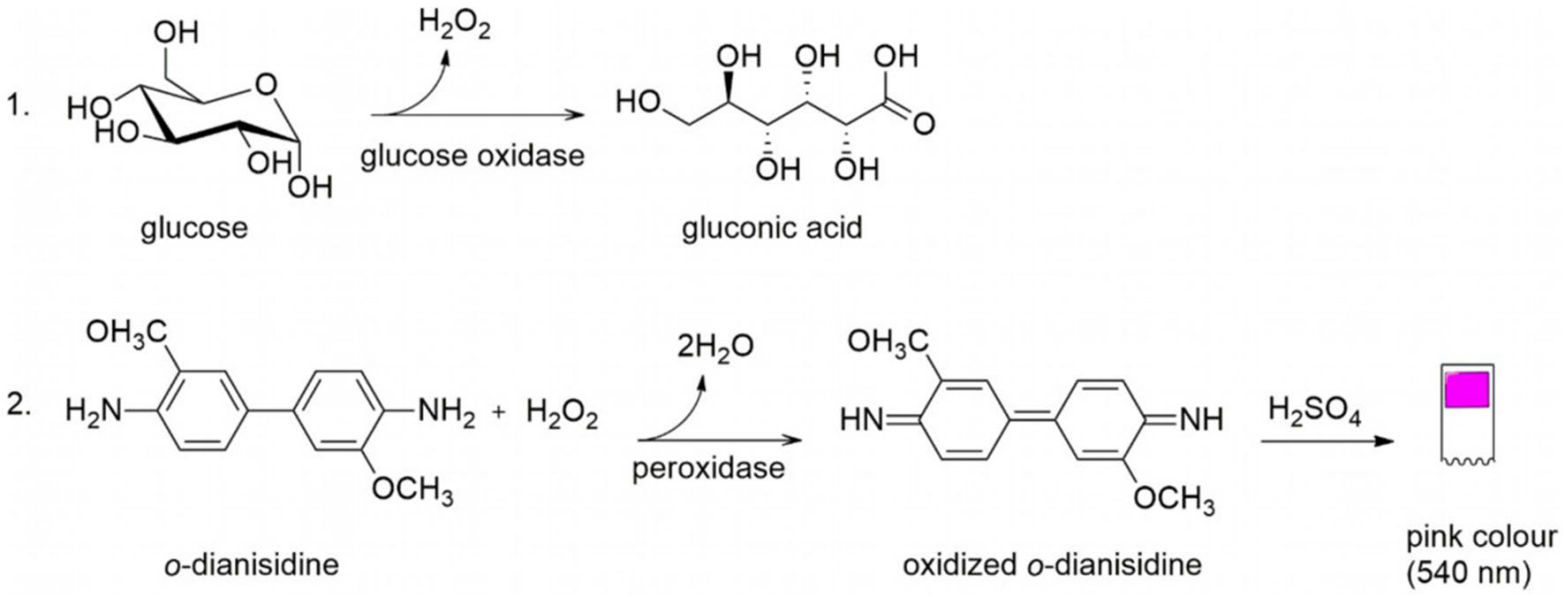
1. Conversion of glucose to hydrogen peroxide and gluconic acid by glucose oxidase. 2. The colorless compound* o*-dianisidine is converted to the oxidized state by peroxidase in the presence of hydrogen peroxide. A red dye is formed by acidification

There are multiple approaches to test for glucose in a dipstick format. The most common method is based on sequential enzymatic reactions. Glucose oxidase (GOD) catalyzes the conversion of glucose into gluconolactone and forms hydrogen peroxide as a side product. In the presence of a peroxidase (POD, e.g., horseradish peroxidase) the hydrogen peroxide reacts with an organic redox indicator yielding a chromophore (Fig. 15). This method is sometimes called the GOD/POD method or test and is preferred over others because of its high specificity for glucose. Alternative strategies often have the challenge of yielding false positive results when galactose or pharmaceuticals are present [66, 67].

A general summary of other common dipstick assays with their corresponding chemical reactions and application purposes is given in Table 3.

**Table 3:** Summary of commercially available paper test strips [68]

Analyte	Method	Application
Aluminum	Aurintricarboxylic acid	Beer processing, juices, food testing, mineral water, industrial water, wastewater
Ammonium	Neßler	Groundwater, surface water, process water, wastewater, agriculture
Arsenic	Modified Gutzeit test	Mineral water, groundwater, surface water, drinking water
Ascorbic acid	Phosphomolybdenum blue	Beer processing, food testing, juices, soft drinks
Calcium	Glyoxal-bis(hydroxyanil)	Beer processing, food testing, juices, milk products, mineral water, soft drinks, boiler water, cooling water, drinking water, industrial water, agriculture
Carbonate	Mixed indicator	Mineral water, aquaculture, drinking water, groundwater, surface water, industrial water, process water
Chloride	Silver chromate	Food testing, groundwater, surface water, wastewater
Chlorine (free)	Redox reaction	Disinfection control, wastewater
Copper	2,2′-Biquinoline	Drinking water, swimming pools, wastewater, electroplating
Formaldehyde	Triazole	Disinfection control, process water
Free fatty acids	pH indicator	Food testing
Glucose	Enzymatic reaction	Beer processing, food testing, juices, milk products, soft drinks
Iron	2,2′-Bipyridine	Food testing, juices, milk products, soft drinks, drinking water, groundwater, surface water, industrial water, wastewater
Lead	Rhodizonic acid	Drinking water, wastewater, agriculture
Manganese	Oxidation/redox indicator	Drinking water, groundwater, surface water, industrial water, wastewater
Nitrate	Modified Griess’ reaction	Food testing, juices, mineral water, aquaculture, drinking water, groundwater, surface water, industrial water, seawater, wastewater, agriculture
Nitrite	Griess’ reaction	Food testing, aquaculture, boiler water, cooling water, drinking water, industrial water, seawater, wastewater
Peracetic acid	Redox reaction	Disinfection control
Peroxidase	Enzymatic reaction	Food testing, milk products
Peroxide	Enzymatic reaction	Milk products, swimming pools, wastewater, disinfection control
Phosphate	Molybdate ion	Food testing, wastewater, agriculture
Potassium	Dipicrylamine	Mineral water, drinking water, industrial water, wastewater, agriculture
Sulfate	Ba-thorin complex	Drinking water, groundwater, surface water, industrial water, wastewater
Sulfite	Nitroprusside/Zn-hexacyanoferrate	Food testing, juices, mineral water, soft drinks, boiler water, cooling water, wastewater
Tin	Toluene-3,4-dithiol	Food testing, juices, milk products, wastewater, agriculture, disinfection control, electroplating
Total hardness	EDTA	Mineral water, drinking water, groundwater, surface water
Zinc	Dithizone	Wastewater, electroplating

### Lateral flow assays

Lateral flow assays (LFA) can be subclassified into lateral flow immunoassays (LFIA) and nucleic acid lateral flow assays (NALFA). LFIAs are protein-based assays relying on antibody–antigen interactions. NALFAs are based on the hybridization of two complementary DNA or RNA strands, making use of a different interaction mode. In the following, the setup and operation mode for the LFIA will be described, as this is the predominant test setup used, but the overall logic principles are similar in both classes.

**Fig. 16:**
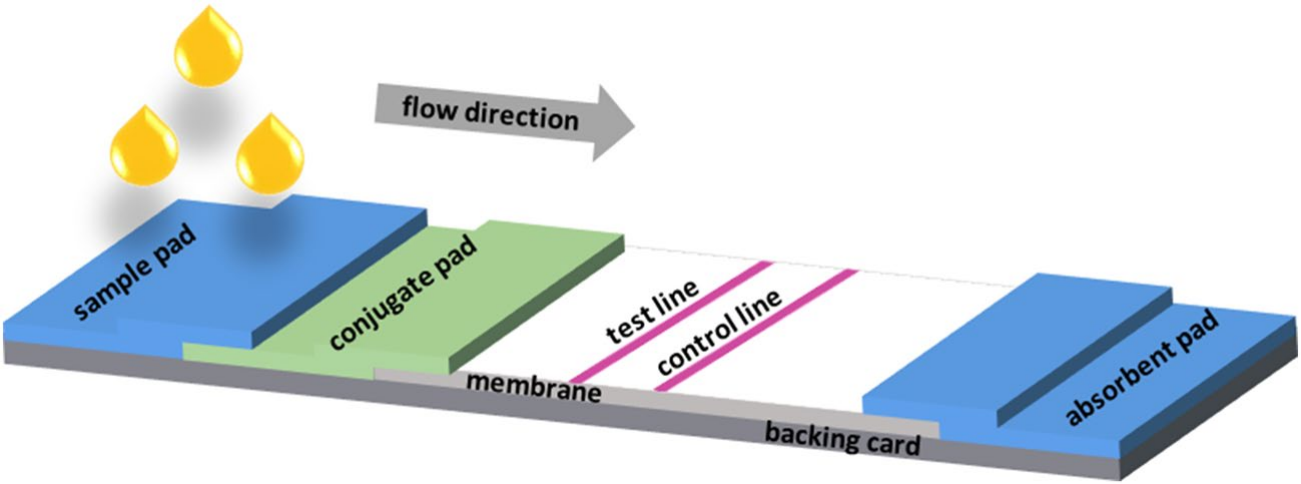
Setup of a lateral flow assay for the detection of one analyte

A classic LFIA consists of different overlapping membranes for sample uptake, conjugation, detection, and fluid aspiration. They all are held together by a backing card, mostly made of a polymeric foil, for better stability and handling. For analyte testing, the sample is applied to the so-called sample pad of highly swellable cellulosic material (Fig. 16). It is absorbent and can contain buffer salts, surfactants, proteins, and certain liquids, to control the flow rate on the one hand, and to prepare the analyte for interaction with the detection system on the other hand. For more complex samples it can also act as a filter for removing redundant material like blood cells.

By capillary forces the sample migrates through the strip by passing through the conjugate pad. This component of the strip holds the detector particles. These are target-specific molecules, like antibodies, that are conjugated to fluorescent or colored particles. The so-called label is required to meet certain terms, some of them being stability in dried state and in solution under various conditions, susceptibility for detection over a large and useful dynamic range, low nonspecific binding, and commercial availability at low cost. The most widely used label are gold nanoparticles, which have an intense purple color, a high stability both in dried and liquid environments, and no need of instrumentation for visualization. Latex microspheres very versatile systems as they can be tagged with a variety of detector reagents, like color or fluorescent dyes, or magnetic and paramagnetic components. Besides, the conjugate pad contains carbohydrates, which act as preservative and resolubilization agents for the conjugate, to ensure a consistent release between individual test strips.

When the analyte is bound to the label, they both migrate to the detection zone on the porous membrane, where specific biological components, like antigens or antibodies, are immobilized in lines to interact with the conjugate-bound analyte and the conjugate itself. The membrane is considered as the most critical element of the LFA and in the majority of cases is made of nitrocellulose. Advantages and disadvantages of this polymeric material, which is essentially not a “paper”, have been outlined above. Important parameters of the membrane are the capillary forces, the ease of binding or immobilizing proteins, and the flow time. The flow time is the time required for the liquid to travel through and completely fill the strip. This is crucial as it defines the time for interaction of the analyte–conjugate and the biomolecules at the test and control line. The test line is responsible for the analyte recognition, while the control line indicates the proper liquid flow throughout the membrane.

The liquid finally flows to the end of the membrane and there into the absorbent pad. This last compartment of the test strip maintains the capillary flow, and prevents backflow by wicking excess fluid. Therefore, it also allows the use of larger sample volumes, which increases the test sensitivity. The readout of either appearing or disappearing lines is performed with the naked eye or a dedicated reader [61].

The assay can be performed in two formats, as a direct, so-called sandwich assay or as a competitive one. The decision of which of the formats can be used depends on the size of the antigen. The direct assay is suitable for all larger analytes/proteins with more than one epitope, meaning a cluster of amino acids, accessible for the specific antibodies, at the same time. The specificity of the antibody–antigen interaction arises from the compatibility of the surfaces of the two species [61, 69]. In a sandwich assay, the antibodies on the label and the test line are complementary and can bind simultaneously to the antigen. The appearance of the test line indicates a positive result.

**Fig. 17:**
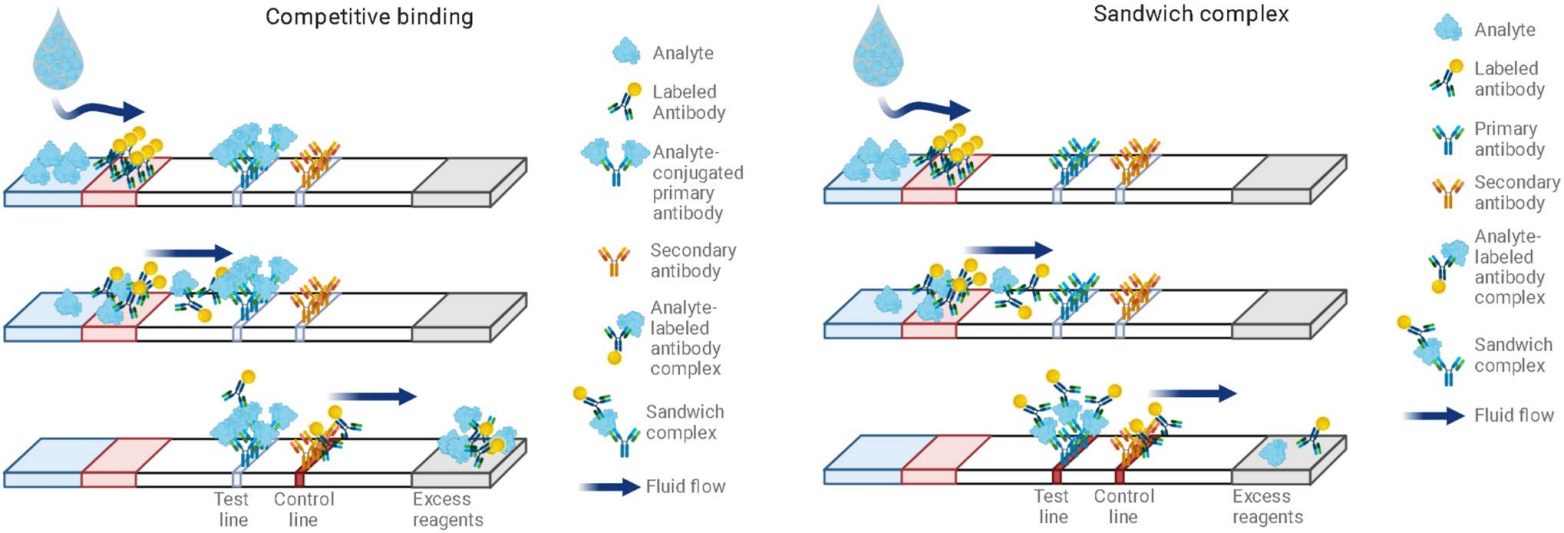
Formats of a LFIA: competitive format (top); sandwich format (bottom). Adapted from [70]. Figure created with biorender.com

The competitive assay format is preferred for targets with relatively small molecular mass or those presenting a single antigen. Here, the antigen itself is immobilized at the test line on the membrane. Hence, no visible line will appear at the test line when the test is positive, meaning the analyte is present in the sample. A schematic depiction is given in Fig. 17 [61].

## Current and future trends in µPAD research

### µPAD fabrication

**Fig. 18:**
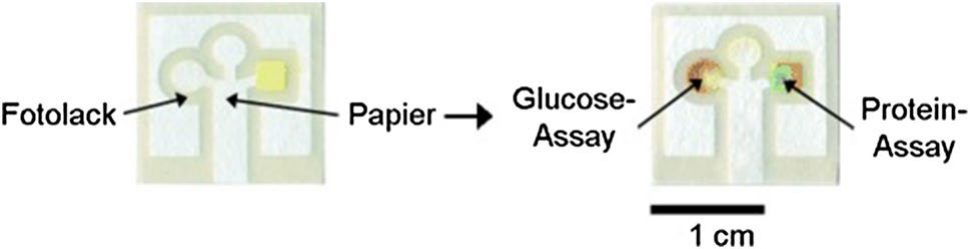
First µPAD for simultaneous glucose (red color change) and protein (blue color change) detection in urine. Reproduced with permission from [71]. Copyright (2007) Wiley-VCH

Extensive research in the µPAD area has been conducted for almost 15 years, since George M. Whitesides and his group introduced the first µPAD device for simultaneous detection of glucose and protein in urine in 2007 (Fig. 18) [71]. Although this might be considered quite a long time in respect of research and possible developments, and a large number of scientific results and demonstrations have been introduced since then, taking a closer look reveals a totally different picture: only a very few examples exist that have been transferred into real field studies up to now [72]. The latter can be considered a major step towards a transfer in real applications.

**Fig. 19:**
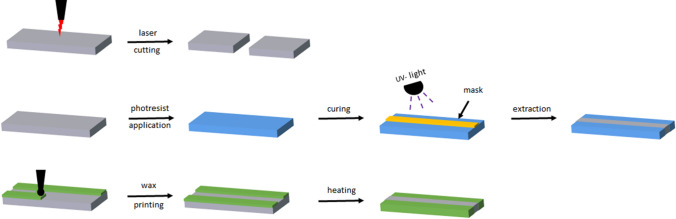
Schematic representation of µPAD fabrication by laser cutting (top), photoresist application (middle), and wax printing (bottom)

Considering µPADs and their possible mass production in the future, two generally different fabrication methods based on two-dimensional shaping (cutting) or hydrophilic/hydrophobic patterning are conceivable (Fig. 19). Two-dimensional shaped assays can be accessed by laser-cutting of the paper, attached to a polymer foil serving as backing for mechanical support [73–77]. On the other hand, µPADs can be fabricated by generating a hydrophilic/hydrophobic pattern on the paper by the deposition of hydrophobic materials (commonly waxes or polystyrene), serving as borders for the fluid front [73, 77–81]. Besides the deposition, spatially resolved chemical modification of the fibers or physical blocking of the pores (mostly by using photoresist agents or polymers as, e.g., polydimethlysiloxane, or photoreactive acrylate and methacrylate polymers [9, 82–85]) are applicable as well [73, 77, 79, 86–89]. All these methods change the wetting behavior of paper by coating the fiber surface or filling of the entire paper cross-section, including the pores [73, 77, 79]. However, differences appear in the spatial resolution and the applicability for mass production. Photoresist materials lead to high resolution patterns, but the procedure is relatively complex. In contrast, cutting, the deposition of hydrophobic agents, and physical pore blocking are more simple procedures; however, resolution of the geometric definition of the channels is lower. Of all the methods, cutting is the simplest procedure with no need for additional agents and chemicals, and therefore one of the most frequently utilized methods for production of µPADs. The fabrication methods presented in this review represent, among all, only a small part of all those reported in the literature. For more information, please also refer to Cate et al. [77]. or Akyazi et al. [73].

### Current and future trends in µPAD research

The properties and applicability for certain analysis of µPADs have been extensively studied in recent years. Despite some outstanding features that have already been mentioned, they still face the following major challenges:

1. Analyte and sample loss due to either adsorptive effects on the cellulose fibers before the test line, evaporation during fluid transport through the device, or insufficient binding kinetics due to a non-optimized capillary flow times.
2. The detection limit of µPADS with classical readout, e.g., colorimetric sensing, is often insufficient for low abundant analytes because of their restricted sensitivity.

LFA tests can have a variety of functionality and signal intensity because of differing paper or (bio-)molecule quality or through varying environmental conditions [73, 79, 90]. As a result of these still unsolved challenges, as of today, µPADs yield only qualitative signals and a quantitative readout is not trivial without any further equipment in the peripheries. One of the most challenging parameters to control in µPADs is the fluid flow through the device. The fluid velocity is an important factor that especially influences the reaction time (kinetics) between the analyte in the fluid front and the functional detector molecule on the paper surface, thus the sensitivity and limit of detection (LOD) of the µPAD are strongly influenced by the capillary flow times (CFT). There are several ways of controlling the CFT, which progress from adjusting the porous structure of the paper itself to inclusion of dissolvable barriers (viscosity modulators) in the channels, or a geometric control of the channel volume. Methods have been outlined in detail in the literature in various recent articles [80, 83, 84, 86, 88, 90–96].

**Fig. 20:**
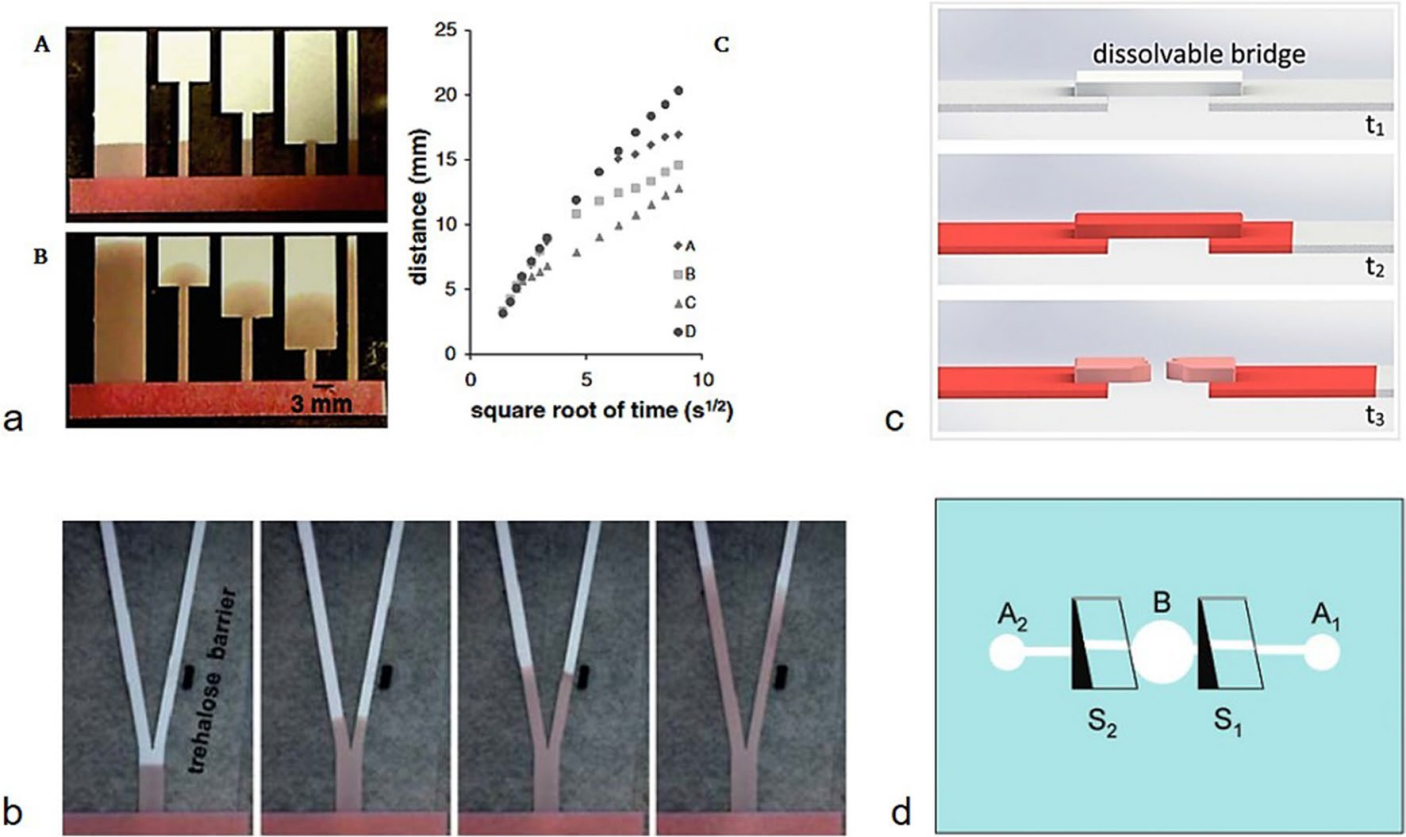
**a** Fluid transport through µPADs with different channel geometries. The fluid front is initially at the same level for all µPADs (up to the time of image A). For an expanding channel, the fluid front slows down in the broader segment compared to if it had continued in the same initial channel width (image B). In graph C, the fluid front distance is plotted vs. the square root of time for the channels A to D. Reproduced with permission from [94]. Copyright (2011) Springer Nature **b** Dissolvable trehalose barrier in the right fork slows down the fluid flow, compared to the left fork with unhindered fluid flow. Reproduced from [95] with permission from the Royal Society of Chemistry. Copyright (2010) Royal Society of Chemistry **c** Operation principle of a dissolvable bridge: the bridge material dissolves to a permanent shut-off state after passing a well-defined volume of fluid at t_3_. Adapted with permission from [96]. Copyright (2013) American Chemical Society **d** Design of a simple two-reservoir (A_1_, A_2_) µPAD with mechanical switches (S_1_, S_2_) that control the fluid flow into the reaction zone (B) Adapted with permission from [97]. Copyright (2008) American Chemical Society

In brief, a simple way to tune the CFT through a µPAD is to change the channel geometry. An enlargement within one microfluidic channel leads to a slowing down of the fluid flow, a narrowing to an increase in fluid velocity (Fig. 20a) [73, 94]. To achieve a particularly high sensitivity while keeping the analysis time to a minimum, a larger CFT before and after the detection zone, as well as a slower CFT value within the detection zone is considered ideal to maximize the rate of analyte binding. In addition, lengthening the paper strip usually has a positive effect on sensitivity, as a higher volume of liquid is transported over the detection zone, which in turn increases the rate of successful analyte binding [90]. Another effective method of slowing down the fluid flow is the incorporation of dissolvable barriers, mostly consisting of sugar, in the channel (Fig. 20b) [95]. In addition, soluble bridge-like structures have also been reported, which connect two parts of the channel and dissolve after a certain amount of fluid has passed through, thus stopping further fluid flow (Fig. 20c) [96]. However, it has to be considered that the deposited chemicals as barrier materials may also interfere with analyte or detection molecules. Furthermore, two-reservoir µPADs with mechanical switches, separating two different fluid sources, were reported. These are connected by two manual switches, enabling timing of the fluid flow in the reaction zone (Fig. 20d) [97].

Finally, CFT can be controlled well by adjusting the porous structure of the paper material itself. With respect to the latter, papermaking technologies are in place to vary, e.g., the pore sizes within the paper sheet. The latter directly affects capillary forces and thereby controls the CFT over a wide range [80].

### Advanced assay designs

**Fig. 21:**
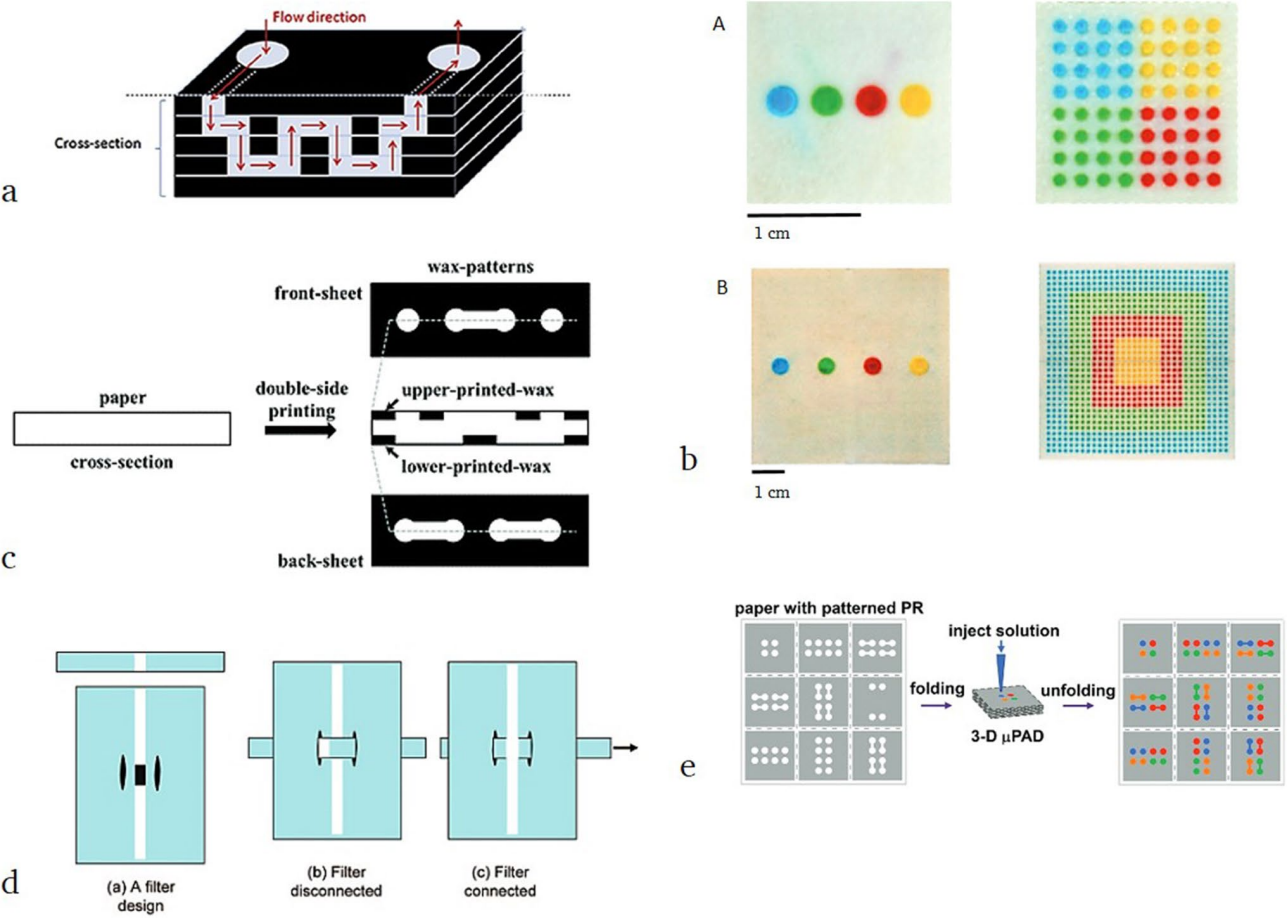
**a** Schematic principle of fluid flow in a 3D µPAD made by hydrophilic/hydrophobic stacked paper layers. Reproduced with permission from [73]. Copyright (2018) Elsevier **b** 3D µPAD that distributes four different sample reservoirs into an array of 64 detection zones (A; 10 µL sample volume per reservoir) and 1024 detection zones (B, 100 µL sample volume per reservoir). Adapted with permission from [98]. Copyright (2008) National Academy of Sciences USA **c** Scheme of the fabrication principle for a one-paper 3D µPAD based on double-sided wax printing. Reproduced from [99] with permission from the Royal Society of Chemistry. Copyright (2015) Royal Society of Chemistry **d** Composition of a µPDA with switchable filter unit that is incorporated by mechanical pulling of the filter strip. Reproduced with permission from [97]. Copyright (2008) American Chemical Society **e** Scheme of “one paper sheet 3D µPAD”, prepared by origami technique. Reproduced with permission from [101]. Copyright (2011) American Chemical Society

A major further development in the field of µPADs was the possibility of building up 3D devices, as first introduced by Whitesides et al. [98]. Initially, 3D µPADs were composed of different stacked hydrophilic/hydrophobic patterned paper layers interconnected by hydrophilic channel parts (Fig. 21a). The additional dimension offered new possibilities in terms of multistep or multiple reactions in one device. Martinez et al. showed the division of four fluid samples of 10 µL each into 64 detection zones and moreover of four fluid samples of 100 µL each into 1024 detection zones (Fig. 21b). Recent research reported the fabrication of 3D µPAD devices out of even one sheet of patterned paper, by adjusting the penetration depth of wax throughout the paper cross-section (Fig. 21c) [99, 100]. Furthermore, efforts are made to incorporate sample preparation steps, e.g., removing disturbing substances or particles from the sample, into the µPAD, to purify/prepare the sample before entering the detection zone. In addition, the integration of filters to remove particles or other disruptive substances, as well as switches in µPADs has been already reported. The separating element is incorporated simply by mechanical pulling and can be disconnected the same way (Fig. 21d) [97].

A further trend in the field is the ongoing simplification of its production. In this context, the work of Liu et al. should be outlined; they described the production of 3D µPADs from a single sheet of paper with folding by hand, a procedure which is called as the origami technique. The device is made from patterned paper that is obtained by a single-step photolithographic modification. They showed a two-analyte assay of glucose and protein in urine on a single origami-fabricated paper 3D µPAD [101].

**Fig. 22:**
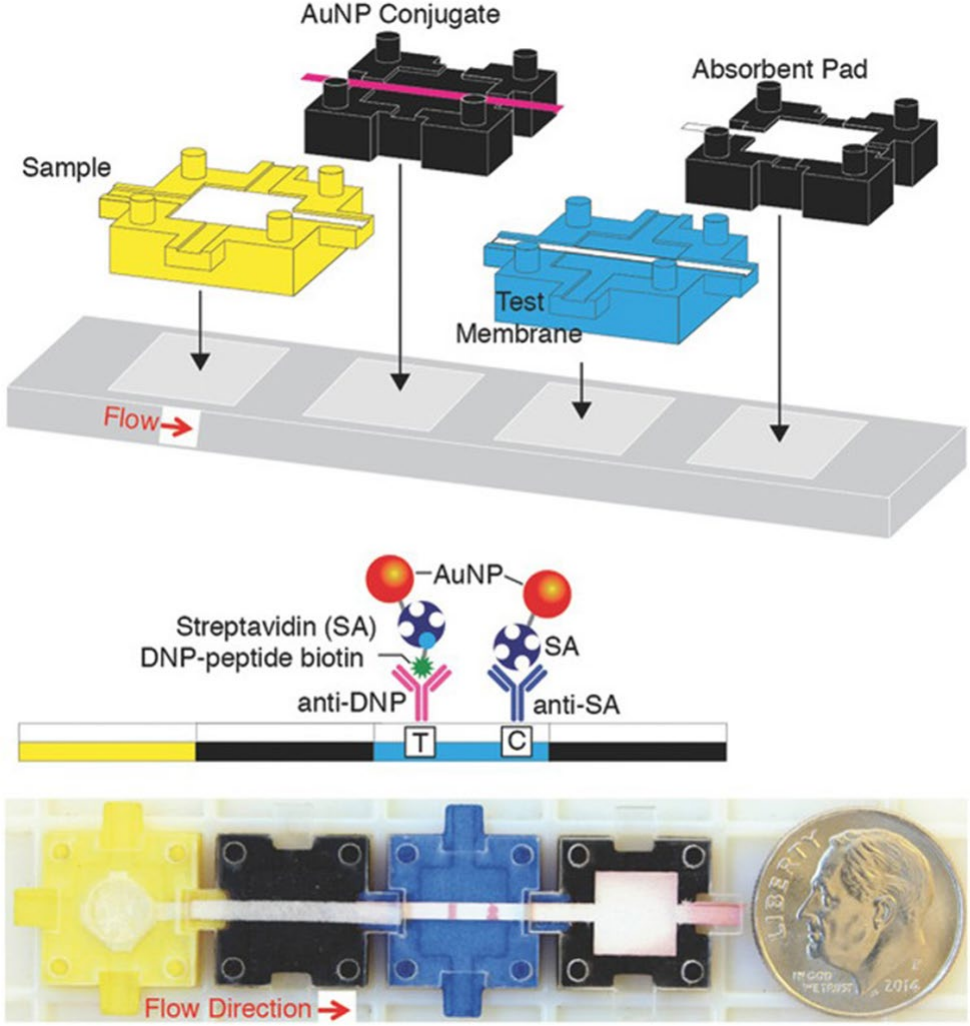
LEGO-like modular assembly for setting up a user-defined lateral-flow immunoassay. Reproduced with permission from [102]. Copyright (2018) Wiley-VCH

The emergence of an increasingly wide range of applications for µPADs recently led Phillips et al. to develop a LEGO-like building block system for µPADs (Fig. 22). The conjugate, sample, test, and absorbent pad can be assembled in any combination as required. This modular building block system enables the production of user-specific µPADs for the analysis of various analytes and samples [102].

**Fig. 23:**
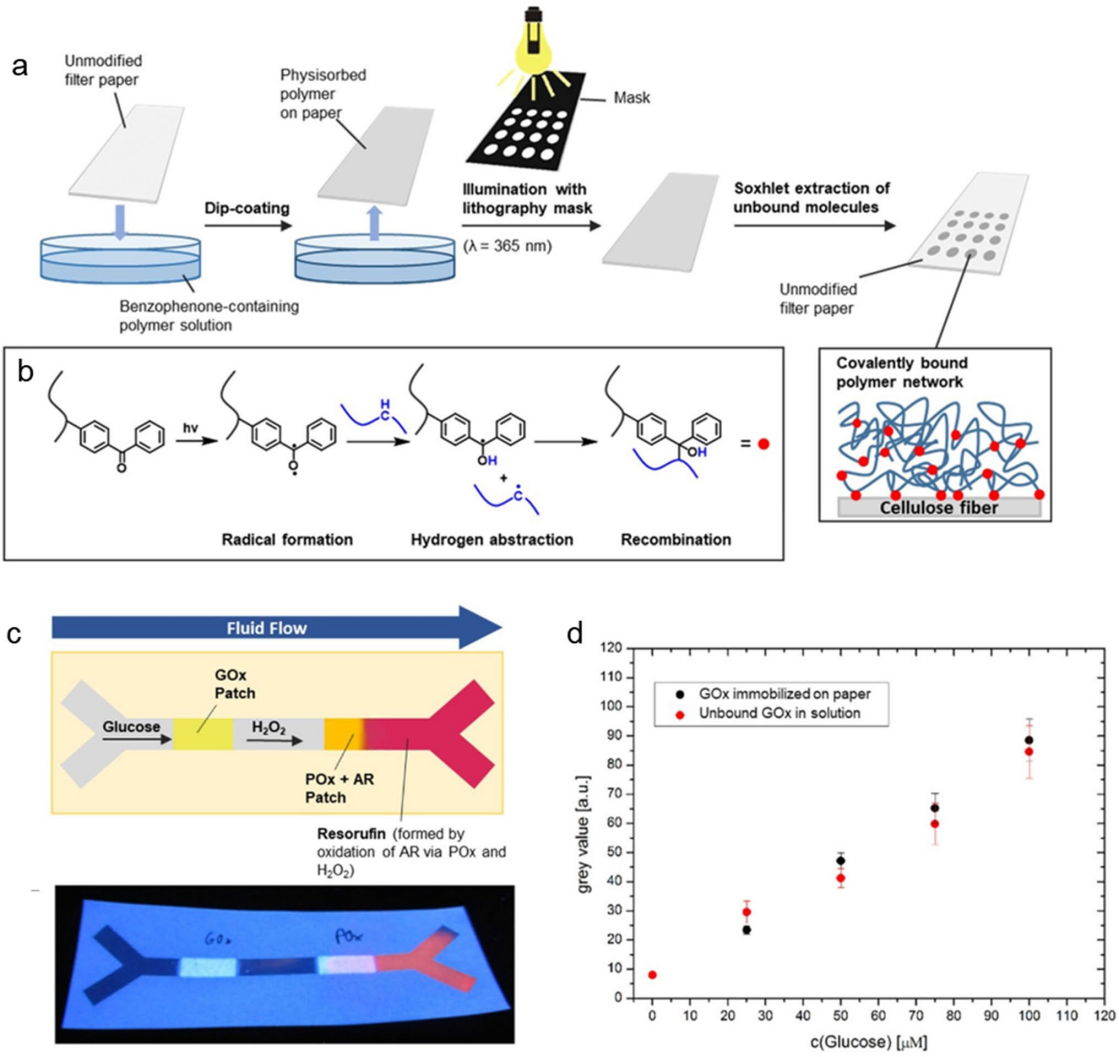
**a** Procedure for the light-induced immobilization of benzophenone-containing polymers on paper fibers by using a photolithography mask. **b** Reaction mechanism of light-induced crosslinking of benzophenone moieties. **c** Schematic of the microfluidic paper-based glucose sensor. The glucose test solution is transported through the sensor by capillary forces only, where it is oxidized to gluconolactone, with hydrogen peroxide as a co-product, as it passes through the glucose oxidase (GOx) patch. Subsequently, hydrogen peroxide oxidizes the non-fluorescent red Amplex dye to red-fluorescent resorufin in the peroxidase (POx) patch. **d** Grayscale fluorescence microscopy values of GOx-modified paper (black) and glucose oxidase in solution (red; solution was dropped onto unmodified paper substrates for examination) plotted against glucose concentration. All figures are reproduced with permission from [85]

Finally, Böhm et al. introduced another simple technique to assemble a colorimetric µPAD glucose sensor, where photoreactive and functional polymers, immobilized on lab-made paper with controllable CFT, were used to immobilize enzymes (Fig. 23). The latter paper-only device was used to detect glucose as a marker, and comparative studies showed that this type of assembly and immobilization of the enzymes did not interfere with enzyme kinetics [85].

## Sensing techniques

The readout of new paper-based analytical devices can be divided in two classes and is based on either an optical or an electrochemical detection system. All of the systems mentioned above rely on optical detection methods, which are among the most inexpensive, simple, and universal methods available [103]. Depending on the nature of the optical signal, the readout can be as easy and cheap as naked eye detection for colorimetric methods [104, 105]. As already mentioned, different lightning conditions or varying visual perception of people can influence the outcome. Therefore, the use of color charts or detectors, among them scanners, cameras, including those of smartphones, can be suitable for the improvement of the readout. None of those devices need special skills to be operated. In particular, smartphone cameras are a promising tool, considering that 67% of the world’s population own these devices, rampant with growing ownership, particularly in the developing world [75]. Images taken of a µPAD could be analyzed by user-optimized software or sent for analysis by a specialist making use of telemedicine [105, 106]. Digital cameras can be used with automatic white balance, which should improve the image quality. But one has to respect that digital cameras and smartphone cameras cannot focus objects which are too close to the lens, whereas office scanners capture images independent of external light sources, ensuring consistent color intensity. The generation of high resolution images, with the µPAD always in focus, is another advantage of scanners which are also widespread devices [105]. Picture analysis can be carried out with standard image analysis or using customized software [75].

**Fig. 24:**
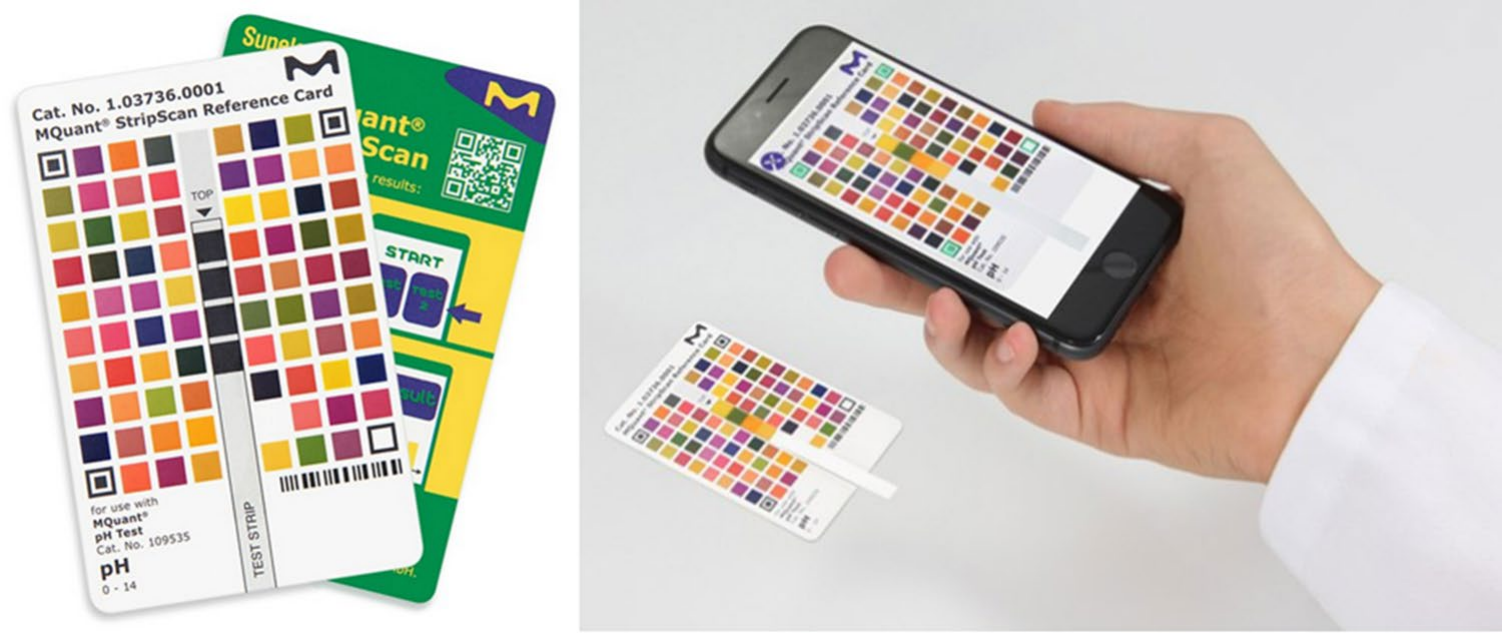
The left image shows a reference card with space in the middle to place the dipstick. The right image shows the dipstick placed in the middle of the reference card and the use of the corresponding smartphone app. Reproduced with permission from [108]. Copyright Merck KGaA, Darmstadt

To overcome the challenges of the readout issues of simple paper strips discussed in “General remarks”, some companies, e.g., Merck KGaA or Hach Lange GmbH, released smartphone apps, free of charge, helping consumers to obtain more precise results from their test strips than the readout by eye. The app analyzes test strips via the smartphone camera. The sample-dipped strip is placed on a credit-card-sized reference card and an image has to be taken with the automatic acquisition mode from the app. The result is displayed on the screen (Fig. 24). This helps one not only to obtain a more precise readout but is also accessible to many users at very low cost, especially when compared to dedicated benchtop readers. It also makes storage and transferring of an analysis result very easy, as the test strip gets digitalized [107].

Besides, more complex devices are used for optical readout. Photoelectric meters or transmittance colorimeters were built that are made of readily available components out of an electronics supply catalogue. Spectrophotometers, fluorometers, microplate readers, photomultiplier tubes, and gel documentation systems are useful, but more sophisticated and rather impractical for point-of-care testing [75].

A major disadvantage of optical sensors is their susceptibility to light, insoluble compounds, and dust. Challenges of this kind can be conquered by the second class of sensor systems, electrochemical detection systems [109]. Those can of course be found in laboratories. However, field measurements and detection can also be carried out with homemade systems, consisting of off-the-shelf units, which are low cost and rather simple. The systems can be designed to be mobile and powered by batteries or by the network, which is especially interesting for their usage in developing countries [110]. The type of measurement, such as voltammetric, amperometric, potentiometric, or conductivity-based, can be adapted to suit the assay and analytes to be tested [75].

**Fig. 25:**
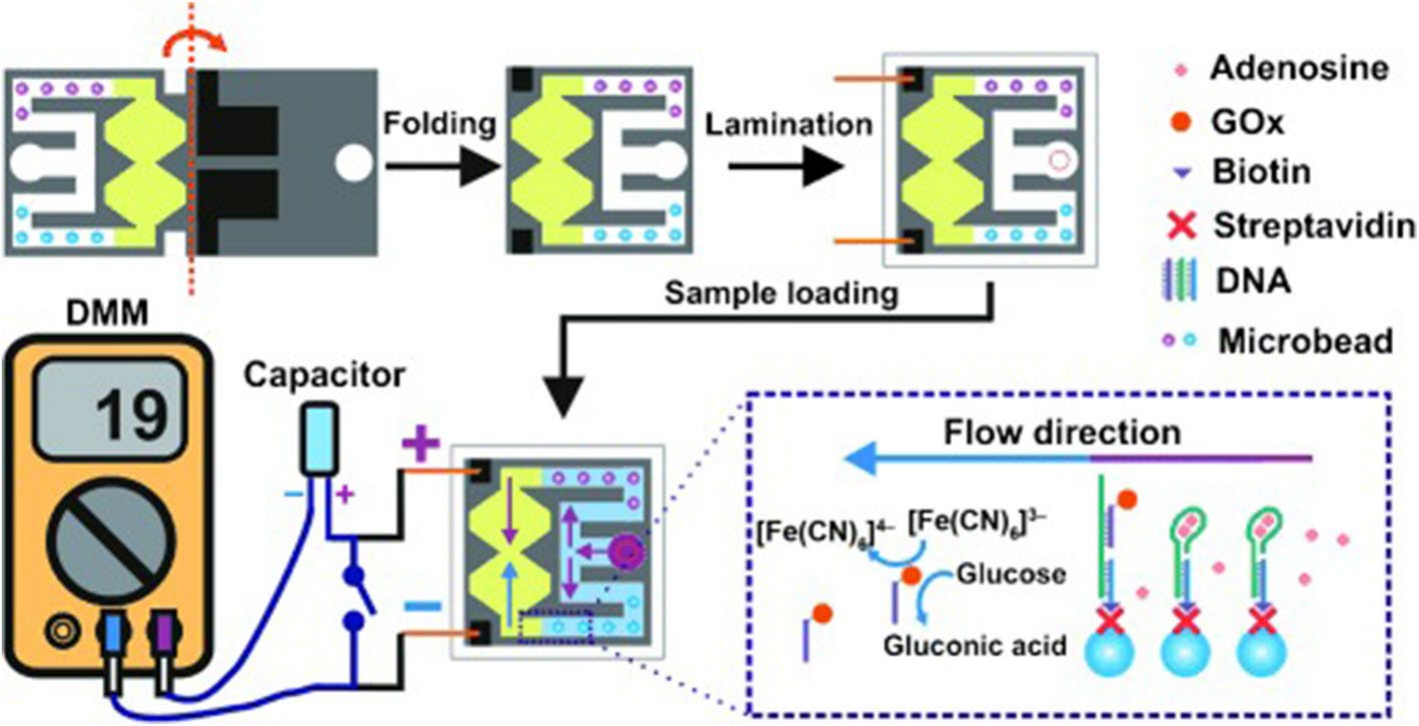
Origami µPAD for adenosine sensing. Paper was wax-printed on the left side, to define an inlet, two hydrophilic channels, and the hourglass-shaped reaction area. There is a thin connection between the two half-cells that acts as a salt bridge. On the right side of the same paper, screen printing was used to define a hydrophilic inlet pattern. Also two carbon electrodes were printed on the right side, which are positioned above the reaction area, when the paper is folded in the middle. The paper is laminated after folding with cutouts to enable a connection of the electrodes and sample application. The reagents were preloaded in the hourglass-shaped pattern. DNA-labeled microbeads are deposited in the hydrophilic channels. An aptamer is loaded in one of the channels. When the sample is applied to the device, DNA-labeled glucose oxidase (GOx) is released downstream when the aptamer binds its target adenosine. The other channel serves as control, where no aptamer is present. The freed GOx catalyzes the oxidation of glucose and thereby the formation of [Fe(CN)_6_]^4−^. The resulting voltage between the two half-cells due to the difference in concentration of both ionic species can be measured with the help of a digital mutlimeter and a capacitor. Adapted with permission from [111]. Copyright (2012) Wiley-VCH

In addition, portable and readily available systems on the market are potential electrochemical detectors for µPADs. Multimeters are available for a reasonable price and some devices can even be connected to smartphones as a data display, storing, and sharing unit [75]. A platform employing a digital multimeter was introduced by Liu et al. [111] (Fig. 25), who built an origami device for adenosine sensing. The device turns into a concentration cell, when adenosine is present, thus generating its own current. They joined up a capacitor between the cell and the digital multimeter (DMM). The capacitor is therefore charged when a current is generated by the concentration cell. By activation of the switch the capacitor is discharged through the DMM. As the current provided by the capacitor is amplified compared to that of a steady measurement, the signal is increased and thereby the sensitivity of the assay is enhanced compared to a setup without a capacitor.

To build the device, a foldable piece of paper was wax-printed on one side, to define an inlet, two hydrophilic channels, and the reaction area having the shape of an hourglass. The thin connection between the two half-cells acts as a salt bridge. On the opposite side, screen printing was used to define a hydrophilic inlet pattern and two carbon electrodes, which are positioned above the reaction area, when the paper is folded. The reagents, glucose and [Fe(CN)_6_]^3−^, were preloaded in the hourglass-shaped pattern. In both hydrophilic channels, DNA-labeled microbeads were deposited. In one channel an aptamer was loaded that binds the incoming target adenosine by releasing DNA-labeled glucose oxidase (GOx) downstream. The other channel serves as control, as no aptamer is present. The freed GOx catalyzes the oxidation of glucose and thereby the formation of [Fe(CN)_6_]^4−^. The resulting voltage between the two half-cells due to the difference in concentration of both ionic species can be measured as described above.

**Fig. 26:**
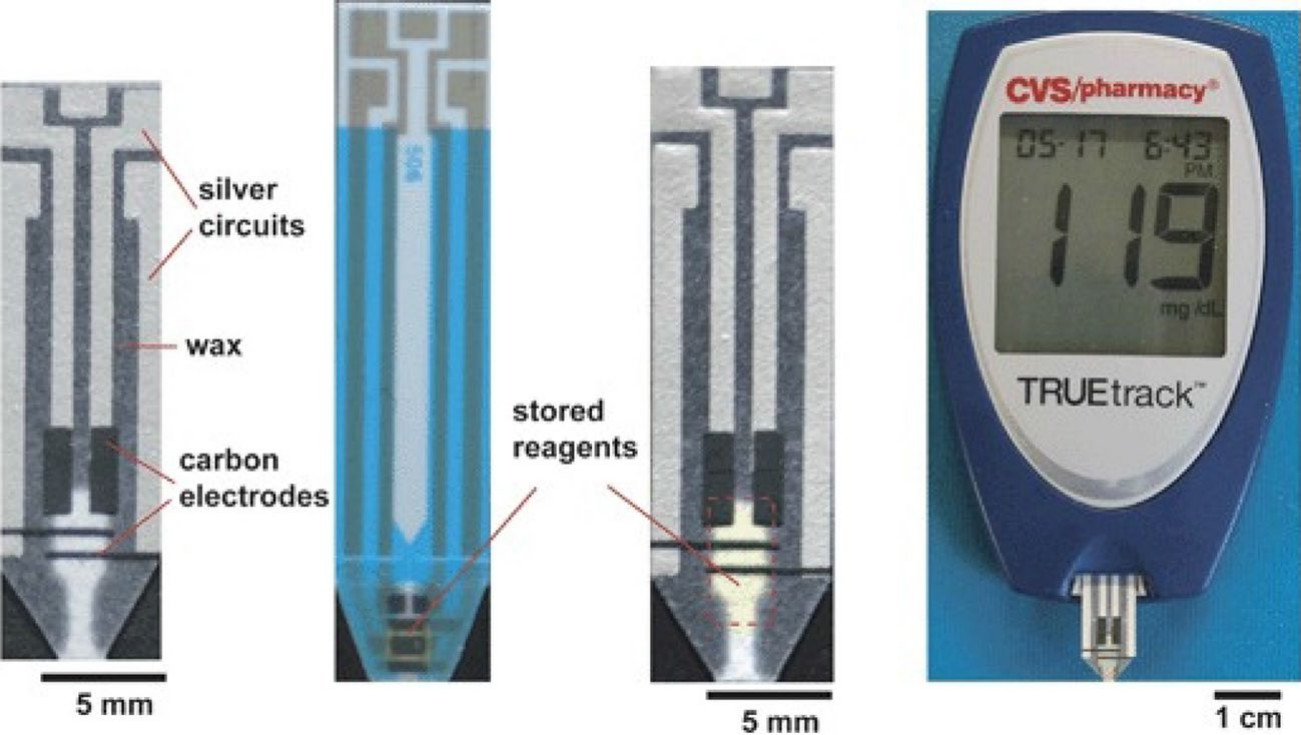
A test strip for complex fluids was developed for the use in combination with a commercial glucometer by Nie et al. [94]. Chromatography paper was modified using wax and screen printing. Reagents were stored in the bottom of the strip. When the sample is applied the compounds migrate through the patterned test strip, allowing the electrical readout with a commercial glucometer. Reproduced from [109] with permission from the Royal Society of Chemistry. Copyright (2010) Royal Society of Chemistry

Moreover, commercial glucometers can be adapted for paper-based sensing, which are designed for use by untrained people. As these devices were introduced several decades ago, development costs were compensated by the market, making the devices affordable [75]. An example of the adaptability of such glucometers was published by Nie et al. [109] (Fig. 26). Chromatography paper was prepared to fit in a glucometer by wax and screen printing of silver wires and four graphite electrodes. The design was inspired by a commercial test strip made from plastic. In general, the glucometer performs amperometric measurements designed to quantify electroactive species. In the classical setup, those species arise from the reaction of glucose with reagents stored in the strip. The group demonstrated that the platform is also suitable for other clinically relevant compounds and complex fluids, such as blood.

## Conclusion/summary

This review summarizes the basic steps to generate functional papers made of cellulose, the world’s most abundant raw material source, beginning with the fundamental chemical properties of cellulose, the possible functionalization strategies, to commercially available functional papers, and the current state of the art of functional papers at the research level. The review also includes a short introduction to papermaking and its history. Thanks to intensive research work, biochemical sensors can be produced that are able to detect specific target analytes in biological samples on paper-based devices. By tuning the fluid flow, the µPAD sensitivity can be enhanced and the detection limit can be lowered. Depending on the assay and fabrication design, they can range from very simple, inexpensive, and being produced in high counts to very sophisticated assay designs with handmade devices. Although already a large number of demonstrations exist to date, there are still major challenges ahead until these types of biosensors will enable medical analysis, address even personalized medical point-of-care issues, and may be used even in remote areas in a broad applicational fashion. With respect to these challenges, answers will to a large extent be given once we start looking more deeply into understanding the highly complex material paper itself. Any successful design of a functional high-tech paper in medical analysis in the future requires a fundamental understanding of the impact of paper’s intrinsic parameters (fiber source, fiber pretreatment, paper porosity, mechanical paper properties, etc.) along different length scales on functionalization, capillary transport, and colorimetric/electrochemical readout with paper-based analytical devices. Glucose test strips and pregnancy tests are most likely just the beginning of the research success with a bright future for these sustainable paper-based point-of-care diagnostic devices.
